# StCoExpNet: a global co-expression network analysis facilitates identifying genes underlying agronomic traits in potatoes

**DOI:** 10.1007/s00299-024-03201-2

**Published:** 2024-04-15

**Authors:** Venkata Suresh Bonthala, Benjamin Stich

**Affiliations:** 1 Institute of Quantitative Genetics and Genomics of Plants, Heinrich Heine University of Düsseldorf, Düsseldorf, Germany; 2 Present Address: Julius Kühn-Institut (JKI), Institute for Breeding Research On Agricultural Crops, Rudolf-Schick-Platz 3a, OT Groß Lüsewitz, 18190 Sanitz, Germany; 3 Max Planck Institute for Plant Breeding Research, Köln, Germany; 4 Cluster of Excellence On Plant Sciences, From Complex Traits Towards Synthetic Modules, Düsseldorf, Germany

**Keywords:** Transcriptome atlas, Co-expression network, *Solanum tuberosum*, Agronomic traits, Tuberization

## Abstract

**Key message:**

We constructed a gene expression atlas and co-expression network for potatoes and identified several novel genes associated with various agronomic traits. This resource will accelerate potato genetics and genomics research.

**Abstract:**

Potato (*Solanum tuberosum* L.) is the world's most crucial non-cereal food crop and ranks third in food production after wheat and rice. Despite the availability of several potato transcriptome datasets at public databases like NCBI SRA, an effort has yet to be put into developing a global transcriptome atlas and a co-expression network for potatoes. The objectives of our study were to construct a global expression atlas for potatoes using publicly available transcriptome datasets, identify housekeeping and tissue-specific genes, construct a global co-expression network and identify co-expression clusters, investigate the transcriptional complexity of genes involved in various essential biological processes related to agronomic traits, and provide a web server (StCoExpNet) to easily access the newly constructed expression atlas and co-expression network to investigate the expression and co-expression of genes of interest. In this study, we used data from 2299 publicly available potato transcriptome samples obtained from 15 different tissues to construct a global transcriptome atlas. We found that roughly 87% of the annotated genes exhibited detectable expression in at least one sample. Among these, we identified 281 genes with consistent and stable expression levels, indicating their role as housekeeping genes. Conversely, 308 genes exhibited marked tissue-specific expression patterns. We exemplarily linked some co-expression clusters to important agronomic traits of potatoes, such as self-incompatibility, anthocyanin biosynthesis, tuberization, and defense responses against multiple pathogens. The dataset compiled here constitutes a new resource (StCoExpNet), which can be accessed at https://stcoexpnet.julius-kuehn.de. This transcriptome atlas and the co-expression network will accelerate potato genetics and genomics research.

**Supplementary Information:**

The online version contains supplementary material available at 10.1007/s00299-024-03201-2.

## Introduction

Potato (*Solanum tuberosum* L.) is a highly heterozygous autotetraploid species and is the world's most crucial non-cereal food crop (Bao et al. 2022). It ranks third in food production after wheat and rice, with an annual global production exceeding 376 million tons (FAO 2021). Biotechnological techniques have gained traction due to the escalating food demand and global climate change, fueled by the expanding human population, to generate better cultivars (Iizumi et al. 2014). To develop improved cultivars, researchers have employed diverse omics approaches, which have been instrumental in augmenting crop productivity (Yang et al. 2021). A milestone in potato omics-based research was the availability of several reference-quality, chromosome-scale and haplotype-resolved genome assemblies, which helped in understanding the complexity and evolution of the potato genome (Potato Genome Sequencing Consortium 2011; Tang et al. 2022; Sun et al. 2022; Hoopes et al. 2022; Bao et al. 2022; Freire et al. 2021; Leisner et al. 2018; Zhou et al. 2020). These potato whole-genome sequencing projects have also contributed to the significant rise in potato transcriptome studies and reported spatiotemporal changes occurring in various potato tissues using RNA-seq (e.g., Massa et al. 2011; Chandrasekar et al. 2022; Tiwari et al. 2020; Pieczynski et al. 2018; Chen et al. 2019; Cao et al. 2020; Tai et al. 2020).

The Potato Genome Sequencing Consortium (2011) reported the sequencing of many tissues of two potato genotypes, such as DM1-3 516 R44 (DM) and RH89-039-16 (RH), under diverse stress conditions. Numerous studies ensued to investigate transcriptional dynamics, such as those covering various biotic and abiotic conditions and cultivars. For example, Massa et al. (2011) used 32 DM RNA-Seq libraries and quantified expression levels of 60% of DM genes under biotic and abiotic stress conditions. Tiwari et al. (2020) investigated the transcriptome of potato tissues generated under varying nitrogen supplies. Their results suggested that the genes from the glutaredoxin gene family, among others, played an important role in conferring nitrogen stress tolerance to potatoes. Chen et al. (2019) analyzed the transcriptional responses upon drought, rehydration and re-dehydration in the drought-tolerant potato landrace Jancko Sisu Yari. They observed that the drought- and rehydration-responsive genes are mainly involved in flavonoid, lipid and sugar metabolism, among others. Chandrasekar et al. (2022) investigated the transcriptional dynamics between resistant and susceptible cultivars against potato cyst nematode (PCN) to identify resistant mechanisms induced by PCN. They identified several disease-resistance genes and transcription factors (TFs) up-regulated in a resistant cultivar (Kufri Swarna).

The availability of plant transcriptomic data in public databases like the Sequence Read Archive (SRA) at the National Center for Biotechnology Information (NCBI) (https://www.ncbi.nlm.nih.gov/sra) has led to the creation of consolidated collections or atlases. These have been developed for several crop species, including *Oryza sativa* (Xia et al. 2017), *Solanum lycopersicum* (Fernandez-Pozo et al. 2017), and *Glycine max* (Machado et al. 2020). These atlases are contributing to understanding the global transcriptional dynamics across cultivars/genotypes/landraces under various stress conditions or between tissues and deciphering the molecular mechanisms that govern biological processes. However, despite the availability of the data at public databases like NCBI SRA from several potato transcriptome studies, an effort has yet to be put into developing a global transcriptome atlas for potatoes.

Housekeeping (HK) genes are those genes expressed relatively stable across all tissue types under various conditions (Czechowski et al. 2005; Bustin et al. 2009)**. **Several of these genes have also been used as internal reference genes in potato real-time quantitative polymerase chain reaction (qPCR) assays. However, many genes considered as HK genes do not exhibit uniform expression across various experimental conditions (Nicot et al. 2005; Hu et al. 2009; Tang et al. 2017). Hence, choosing appropriate reference genes is critical in potato qPCR assays. With the emergence of next-generation sequencing technology, RNA-Seq data can be used to evaluate commonly used reference genes and propose new ones (Yim et al. 2015; Machado et al. 2020). Although numerous transcriptome datasets are available at public repositories such as NCBI SRA from several potato transcriptome studies, no attempt has yet been made to assess the commonly used reference genes and identify new ones to improve the precision of potato qPCR assays across various experimental conditions.

Tissue-specific (TS) genes are those expressed and function in a specific tissue preferentially over the other tissues. Identifying these genes helps better understand tissue-gene relationships (Xiao et al. 2010). For example, the combinatory action of MADS and AP/ERF family transcription factors regulates the development of distinct floral parts in *Arabidopsis thaliana* (Chi et al. 2017). Machado et al. (2020) identified several TS genes specific to nodules, endosperm and flowers in soybean using many RNA-Seq datasets. Despite the availability of numerous RNA-Seq datasets, a systematic identification of TS genes in potato is lacking.

Gene co-expression networks (GCN) provide a robust method to explore transcriptomic data. These networks are undirected graphs of nodes that correspond to genes and are interconnected by edges based on significant co-expression between them, representing transcriptionally coordinated genes often involved in the same biological process (Stuart et al. 2003). GCNs are effective tools in functional genomics as they enable the inference of putative gene functions and regulatory mechanisms through gene co-expression (Ballouz et al. 2015). Additionally, GCNs permit the simultaneous identification and classification of numerous genes with similar expression patterns (Serin et al. 2016). For example, GCNs have been employed in specific areas of plant research, such as investigating the genetic basis of plant natural products (Wisecaver et al. 2017), nitrogen metabolism for plant growth (Gaudinier et al. 2018), cell wall development (Rao et al. 2019), and resistance responses to powdery mildew (Zhang et al. 2016a). GCNs have been constructed and explored gene co-expressions to understand the transcriptional regulation of various biological processes in several plants, such as *Arabidopsis thaliana* (Burks et al. 2022), *Oryza sativa* (Sircar et al. 2022), *Zea mays* (Yu et al. 2017), *Hordeum vulgare* (Lee et al. 2020), and *Glycine max* (Almeida-Silva et al. 2020).

A few GCNs have also been constructed for potatoes using publicly available transcriptomic datasets. Massa et al. (2011) constructed a GCN using RNA-Seq data from 32 DM libraries. They identified 18 co-expression clusters, representing genes with highly correlated expression profiles in a biological process. Ramšak et al. (2018) constructed a GCN using two microarray datasets to understand immune signaling in potatoes better. They discovered a link between ethylene (ET) and salicylic acid (SA) signaling pathways. Specifically, they found that activating the ET signaling module via the Ethylene Insensitive3 gene triggers the expression of the Nonexpressor of PR Genes1, a critical regulator of the SA pathway. Yan et al. (2018) constructed a GCN using 16 RNA-Seq datasets covering 11 cultivars to investigate the resistance of potatoes. This GCN analysis revealed that 134 genes were significantly enriched and exhibited high levels of co-expression in Andigena, particularly concerning potato disease and stress resistance. This finding highlighted the significant impact of evolutionary pressures during artificial potato domestication. In addition, several studies used GCNs to investigate transcriptional regulation under various stress conditions using datasets generated in respective studies. Qin et al. (2022) unraveled cultivar-specific rooting depth responses to drought stress in potatoes using GCN. Despite the growing availability of gene expression datasets (RNA-Seq) that provide unbiased representations of gene expression patterns across various potato cultivars worldwide, the GCN analyses conducted so far have focused on case–control experiments to address specific objectives or have used small datasets. This limited approach has hindered the ability to uncover the global transcriptional landscape of potatoes in different tissues and conditions.

The objectives of our study were to (i) construct a global expression atlas for potatoes using publicly available transcriptome datasets, (ii) identify housekeeping and tissue-specific genes, (iii) construct a global co-expression network and identify co-expression clusters, (iv) investigate the transcriptional complexity of genes involved in various essential biological processes related to agronomic traits, and (v) provide a web server to easily access the newly constructed expression atlas and co-expression network to investigate the expression and co-expression of genes of interest.

## Materials and methods

### Potato genome and annotation data

We used the genomic sequence and annotation data for the potato reference genome, dAg, from our recent study (Bonthala and Stich 2022). The gene annotation contained 39,088 and 53,352 genes and transcripts, respectively. We used the gene annotation’s exon–intron boundaries (gff3 format) as a reference guide in read mapping. From the annotation data, we used functional annotation such as gene description, gene ontology (GO) terms, Pfam domains, InterProScan descriptions, and Arabidopsis ortholog descriptions.

### Potato RNA-Seq data, processing and quality control

We searched the NCBI SRA database (https://www.ncbi.nlm.nih.gov/sra) for potato transcriptome datasets. We exported the metadata using Run Selector (as of June 2022) with the following parameters: AssayType: RNA-Seq, LibrarySource: TRANSCRIPTOMIC, Organism: *Solanum tuberosum*, Common name: potato, and Platform: Illumina and BGISEQ. In addition, we also searched extensively for additional potato transcriptome datasets in the literature (as of June 2022), and we added the metadata of new datasets to exported metadata of NCBI. Using this metadata, we downloaded experiment details using NCBI e-fetch (Leinonen et al. 2010). Using these experiment details, we excluded samples showing technical issues, such as empty FASTQ files, pair-end samples with single-end reads, and pair-end samples with an unequal length of reads. Finally, we downloaded 3,227 SRA files and converted them into FASTQ files using SRA-TOOLKIT v3.0.0 (Leinonen et al. 2010). We performed the quality assessment of FASTQ using FastQC v0.12.1 (Andrews 2010). We removed the low-quality reads, i.e., those with an average base quality of less than 20 or containing adapter sequences, using Trimmomatic v0.39 (Bolger et al. 2014). We inferred the library strandedness for each sample by applying the approach presented by Zheng et al. (2019). This approach involves mapping 100,000 reads for each sample using Kallisto  v0.46.1 (Bray et al. 2016) onto dAg genome under all three library types (–rf-stranded, –fr-stranded and none) separately, followed by comparing the obtained results across all three library types.

### Transcript assembly and gene expression quantification

We aligned the high-quality reads of each library to the potato reference genome (dAg) using HISAT2 v2.2.1 (Kim et al. 2019) based on the default parameters. The log files were processed to obtain read mapping statistics. We performed transcript assembly and quantification of gene expression using StringTie v2.2.1 (Pertea et al. 2015) as follows: (1) The mapped reads in bam format were assembled into transcripts using StringTie for each sample with the following parameters: at least five reads supporting exon-junction boundary (-j 5), average read depth for a transcript of at least 10 (-c 10), and the inferred library strandedness was considered. (2) Merging of assembled transcripts into tissue-wise separately for each of 15 tissues using stringtie-merge with the following parameters: minimum transcript length of 200 bp (–m 200) and minimum isoform fraction of 0.5 (–f 0.5). (3) Finally, transcriptome assemblies from each of the 15 tissues were merged into a single non-redundant transcriptome assembly using stringtie-merge with the earlier parameters. (4) Normalized expression was estimated in TPM using stringtie with the -e option for each sample. In addition, raw read counts for each gene were calculated using the prepDE.py3 script (Pertea et al. 2015). Finally, Gffcompare v0.12.6 (Pertea and Pertea 2020) was used to compare the above-generated non-redundant transcriptome assembly with the reference transcripts (dAg).

### Sample clustering

We assessed the sample clustering patterns by submitting genes with mean log2 (read count + 1) >  = 1 to hierarchical clustering based on Pearson’s correlation matrices using R. We inspected the resulting tree for mislabeled samples.

### Identification of novel genes and splicing isoforms

We relied on the Gffcompare v0.12.6 (Pertea and Pertea 2020) output files to identify novel genes and isoforms. Transcripts not overlapping with known reference transcripts were assigned to class U. The nucleotide sequences of the class U transcripts were extracted and translated using TRANSDECODER v5.7.0 (Haas et al. 2013). We predicted protein domains using HMMER v3.3.2 (Finn et al. 2015) with default parameters and the PFAM database v35 (Finn et al. 2016). We performed functional annotation using AHRD for class U transcripts (https://github.com/groupschoof/AHRD). We classified class J transcripts as putative novel isoforms.

### Identification of housekeeping and tissue-specific genes

We used the data of 15 tissues to identify housekeeping (HK) genes in potato and assessed the variability in gene expression of HK genes using the approach of Hoang et al. 2017. The approach involves the following criteria: each gene is classified as expressed if TPM >  = 1 in at least one sample or otherwise not expressed. We calculated the mean TPM of genes expressed in all samples by taking the average gene expression across all samples, followed by computing the Coefficient of Variation (CoV). We calculated the ratio of the maximum to minimum (MFC) by dividing the largest by the smallest TPM values, followed by computing a product score (MFC-CoV) based on the product of CoV and MFC for each gene. Finally, we classified genes with MFC-CoV scores within the first quartile as HK genes.

We used the log2 transformed TPM values to identify tissue-specific (TS) genes. All 15 tissues were compared against each other to find significantly overexpressed genes using LIMMA v3.58.1 (Ritchie et al. 2015). We considered genes with log2 (fold-change) >  = 2 with adjusted *p* <  = 0.05 as significantly overexpressed. If gene G was overexpressed in tissue T compared with all other tissues, then gene G was considered specifically expressed in tissue T. Further, we assessed the tissue-specific expression of HK and TS genes using the Tau index as previously described. The Tau values scale from 0 to 1, where low and high values indicate widely expressed and more tissue-specific genes, respectively (Kryuch-kova-Mostacci and Robinson-Rechavi 2017).

### Identification of genes encoding transcription factors and nucleotide-binding and leucine-rich repeats

We identified transcription factors (TFs) by feeding the protein sequences of the longest isoform of 39,088 genes to iTAK v1.7a (Zheng et al. 2016) in dAg. Using the NLR-Annotator v2, we identified the nucleotide-binding and leucine-rich repeat (NLR) encoding genes (Steuernagel et al. 2020).

### Potato orthology map

We used the protein sequences of the longest isoform of the eight potato clones, for which chromosome-scale genome assemblies are available (Table S1), and fed them to OrthoFinder (Emms and Kelly 2019) to compute orthogroups across eight potato clones.

### Network reconstruction, module detection and gene ontology enrichment

We constructed a Pearson correlation coefficient (PCC) based co-expression network for all genes expressed in at least one transcriptome library with a TPM of 1 using the pcc.py script of LSTrAP v1.3 (Goh and Mutwil 2021). We converted the PCC-based co-expression network into a Highest Reciprocal Rank (HRR) co-expression network using parameters of a maximum HRR of 50 and a PCC cut-off of 0.5 with a second-level neighborhood. We clustered the HRR co-expression network to detect co-expressed modules using the heuristic cluster chiseling algorithm (Mutwil et al. 2010) with default parameters. We performed gene ontology enrichment for each of the detected co-expression modules. We used the CoNekT v1.1.1 framework (Proost and Mutwil 2018) for network reconstruction, module detection and gene ontology enrichments. Finally, we developed a web server to easily access the expression atlas and the co-expression network by adopting the CoNekT framework due to its rich features (Proost and Mutwil 2018).

### Identification of homologs in the reference genome

Using BLAST (Altschul et al. 1990), we identified homologs in the reference genome (dAg) for a selected set of potato genes. We considered the first best hit as the homolog for respective genes. We used the CDS sequences of 14 Rpi genes (Armstrong et al. 2019) and the protein sequence of PhAN2 (UniProt ID: A4GRV2) (Laimbeer et al. 2020) to identify their homologs in the reference genome. We used the protein sequences of the tuber identity gene (IT1; ID: Soltu.DM.06G025210) and the SELF-PRUNING 6A (SP6A; ID: Soltu.DM.05G026370) genes to identify respective homologs in the reference genome (Tang et al. 2022). We used the CDS sequences of eight S-RNases involved in self-incompatibility, as mentioned in Dzidzienyo et al. (2016), to identify their homologs in the reference genome.

## Results

### Data collection, processing, mapping of reads and expression quantification

We performed extensive literature mining to gather as many potato RNA-Seq datasets as possible. We downloaded 3227 raw read sequencing files (.sra) from the National Center for Biotechnology Information (NCBI) Sequence Reads Archive (SRA) database and converted them into FASTQ format. We combined reads obtained from the same library in a single FASTQ file for single-end (SE) data or two files for paired-end (PE) data, resulting in 2,636 libraries (85.24% are PE and 14.75% are SE data) from 155 NCBI BioProjects comprising 20 broad tissue categories (Table S2).

We excluded reads containing adapter sequences or reads with an average quality of less than 20. We excluded 32 samples that contained less than 100,000 reads or for which less than 50% of reads remained after trimming. The reads from each sample were mapped onto the reference genome, followed by assembling transcripts and then performing quantification of transcript abundance. We used 2.604 samples containing an average of 23,060,781 read pairs per sample with PE data and 36,832,773 reads per sample with SE data for read mapping. Mapped and uniquely mapped reads corresponded to an average of 80.73% and 67.80%, respectively. We excluded 157 samples in which >  = 50% of reads failed to map or >  = 40% could not map uniquely. Finally, we excluded 106 samples which were made of combinations of multiple tissues, such as callus, plantlet, seedling, whole plant and mixed tissues. In total, we kept 2341 samples from 147 NCBI BioProjects for downstream analyses (Table S3).

Leaf was the most abundant tissue representing 45.4% of the samples, while petiole tissue represented 0.21% (Table S4). We have also found that about 58% (1361 of 2341) of the libraries were unstranded. Finally, we assembled transcripts and estimated transcript abundances in raw read counts and transcripts per million (TPM) at the gene level (Figure S1).

### Systematic analysis of thousands of potato RNA-Seq samples

**Fig. 1:**
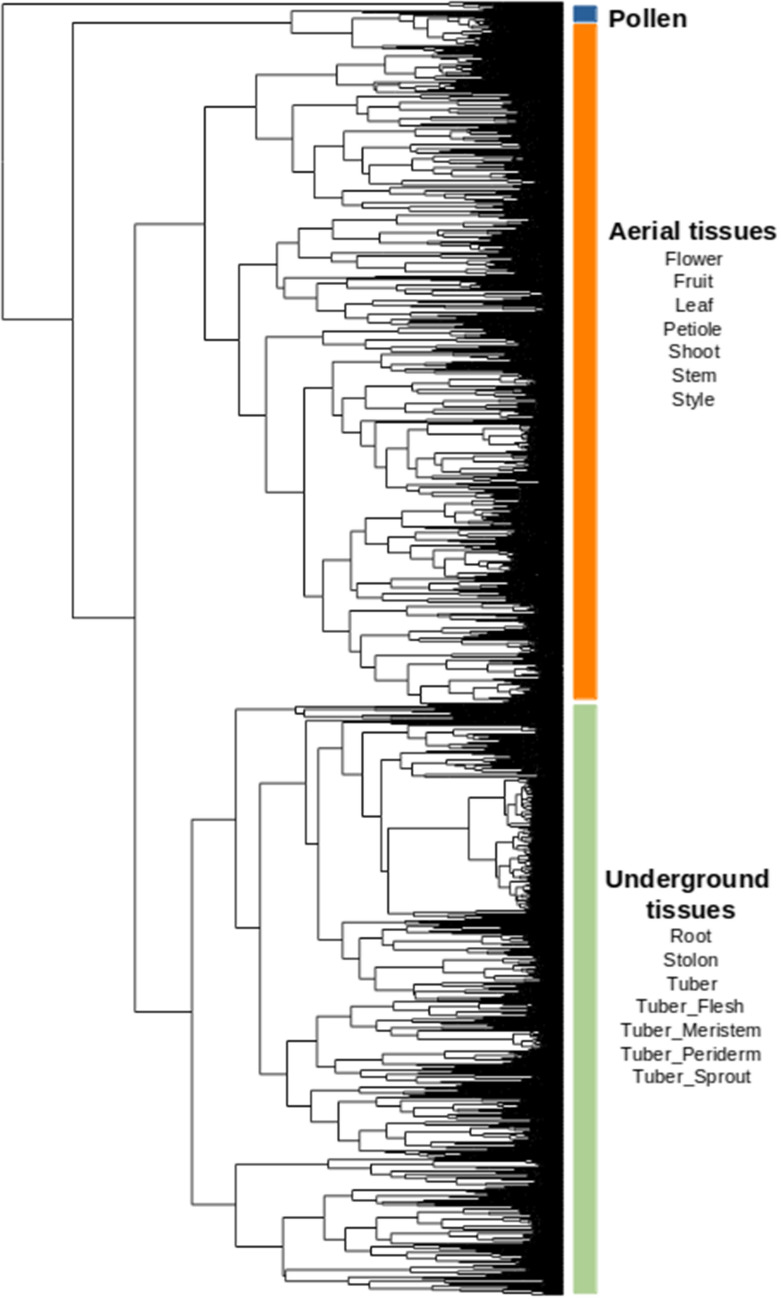
Hierarchical clustering of samples using their transcriptional profiles. Raw read counts were used to compute Pearson's correlations, followed by hierarchical clustering in R. Samples were grouped into three major clades, i.e., aerial, underground tissues, and pollen clusters. A total of 42 samples were mislabeled. For e.g., 18 samples of underground tissues were grouped with samples of aerial tissues

In transcriptomics studies, the clustering of samples is instrumental in identifying broad transcriptional similarities between samples and identifying potential technical artefacts and mislabeled samples. Here we employed hierarchical clustering to identify mislabeled samples. The clustering analysis revealed two major clades comprising samples from aerial and underground tissues. However, interestingly, we found an additional cluster consisting of samples from pollen only (Fig. 1). In addition, we observed that seven samples from underground tissues were clustered with aerial tissues, while 35 aerial tissues clustered with underground tissues. In order to avoid the influence of these potentially mislabeled samples, we excluded these 42 samples from the downstream analyses.

In this study, we classified a gene as expressed if the gene had a minimum TPM threshold of 1 in at least one sample and found that across all samples about 87% of known potato reference genes (33,981 of 38,977) were expressed. An average of 18,589 genes were expressed per sample. The tissue with the highest number of expressed genes was leaf (31,427 genes), whereas pollen had the lowest number of expressed genes (12,801 genes) (Table S5). We found that 12,600 genes were expressed in at least 90% of samples, including 1121 genes in all 2299 samples. About 83% of all genes not expressed in any sample had coding sequences comprising < 300 codons (Figure S2).

### Housekeeping and tissue-specific genes

**Fig. 2:**
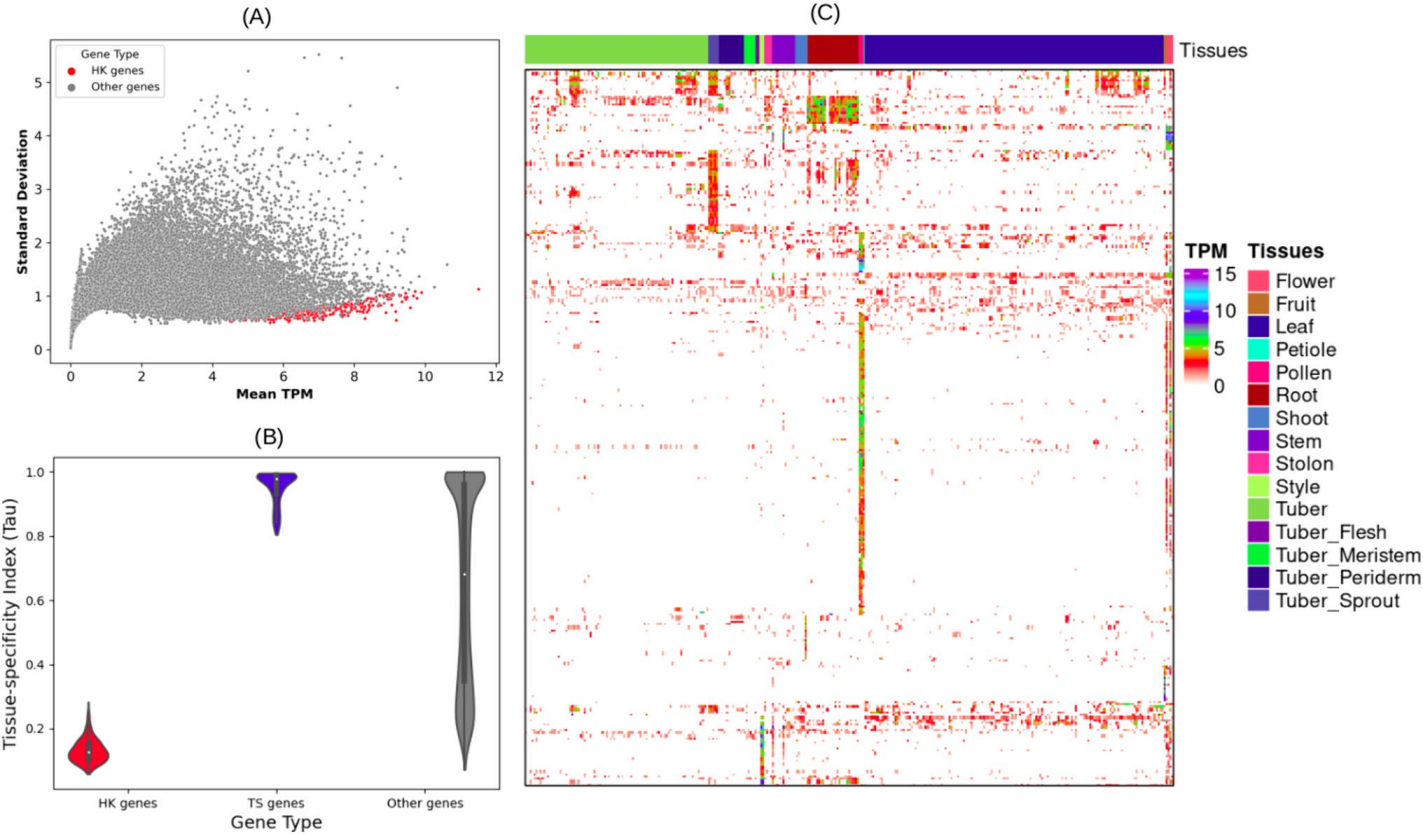
Housekeeping (HK) and tissue-specific (TS) genes. **A** Scatter plot of mean versus standard deviation of transcripts per million (TPM) showing uniform and stable expression of 281 HK genes. Grey dots represent all the non-HK-expressed genes (TPM ≥ 1 in at least one sample). **B** Violin plot of the distribution of Tau indexes for housekeeping (HK), tissue-specific (TS), and the other genes. Low Tau values indicate a stable and constitutive expression, while higher values support tissue-specificity (Tau > 0.8). **C** Global transcriptional patterns of tissue-specific (TS) genes. Expression values are represented as log2 transformed transcripts per million (TPM) values in all tissues (2299 samples)

Due to the availability of an extensive collection of RNA-Seq samples covering a wide range of tissues and environmental conditions, we also pursued identifying housekeeping (HK) genes for potatoes. In this study, we identified 281 HK genes (Table S6) using a previously described method (Hoang et al. 2017). We evaluated the expression levels of HK genes in all tissues and found that the genes had very low expression variation (Fig. 2A). Furthermore, we used the tissue-specific index Tau to estimate tissue-specificity and confirm whether the identified HK genes broadly expressed across all tissues. The Tau scores of the HK genes ranged from 0.058 to 0.282 (Fig. 2B).

We compared the global expression patterns between tissues to identify tissue-specific genes (Figure S3). All 15 tissues were compared pairwisely, resulting in 308 genes with a significantly higher expression in a single tissue compared with all the others (Fig. 2C and Table S7). Interestingly, more than 90% (278 of 308) of these genes had Tau indexes > 0.8 and a median Tau of 0.97005 (Fig. 2B). Given their solid preferential expression in particular tissues, we called these genes tissue-specific (Tau > 0.8). The tissue-specific genes ranged from 11 in roots to 137 in pollen. Interestingly, 18 tissue-specific genes belonged to ten transcription factor (TF) gene families (Table S8). The number of tissue-specific TF genes ranged from one in fruit, root and style to nine in pollen.

### Identification of novel transcripts

We compared the genomic coordinates of the transcripts assembled in our study with the reference transcripts (dAg) using Gffcompare (Pertea and Pertea 2020) and categorized them into 15 classes (Table S9). We found that 99.22% (58,274 of 58,734) of the transcripts precisely matched the exon–intron splice junctions of known transcripts (class “ = ”). We also investigated class-J and class-U categories, which account for 17,312 and 30,832 transcripts, respectively. Class-J comprises multi-exon transcripts with at least one known exon junction, while class-U encompasses transcripts located in intergenic regions. While class-J transcripts include new isoforms of known genes, those from class-U identify potentially new genes. We found that approximately 84% (14,476 of 17,312) of the class-J transcripts and about 11% (3489 of 30,832) of the class-U transcripts contain a complete open reading frame (ORFs) (Table S9). We found that 14,476 class-J transcripts belong to about 30% of reference genes (11,736 out of 39,088). In addition, we found 608 transcription factors belonging to 59 TF families (Table S10) and 94 NLR genes (Table S11) in class-J transcripts. The gene ontology enrichment analysis revealed that the class-J transcripts were enriched with several biological processes (FDR < 0.05). The top five enriched biological processes were “response to abscisic acid” (GO:0009737), “salt stress (GO:0009651)”, “water deprivation” (GO:0009414), “cold response” (GO:0009409), and “positive regulation of transcription, DNA-templated” (GO:0045893) (Table S12). On the other hand, we found 1150 non-transposon genes within 3489 class-U transcripts. Interestingly, we found 108 transcription factors (TF) belonging to 26 families (Table S13) and five NLR genes (Table S14) in the class-U transcripts. However, we did not find significantly enriched gene ontology terms in these transcripts.

### Co-expression network construction and detection of co-expression clusters

**Fig. 3:**
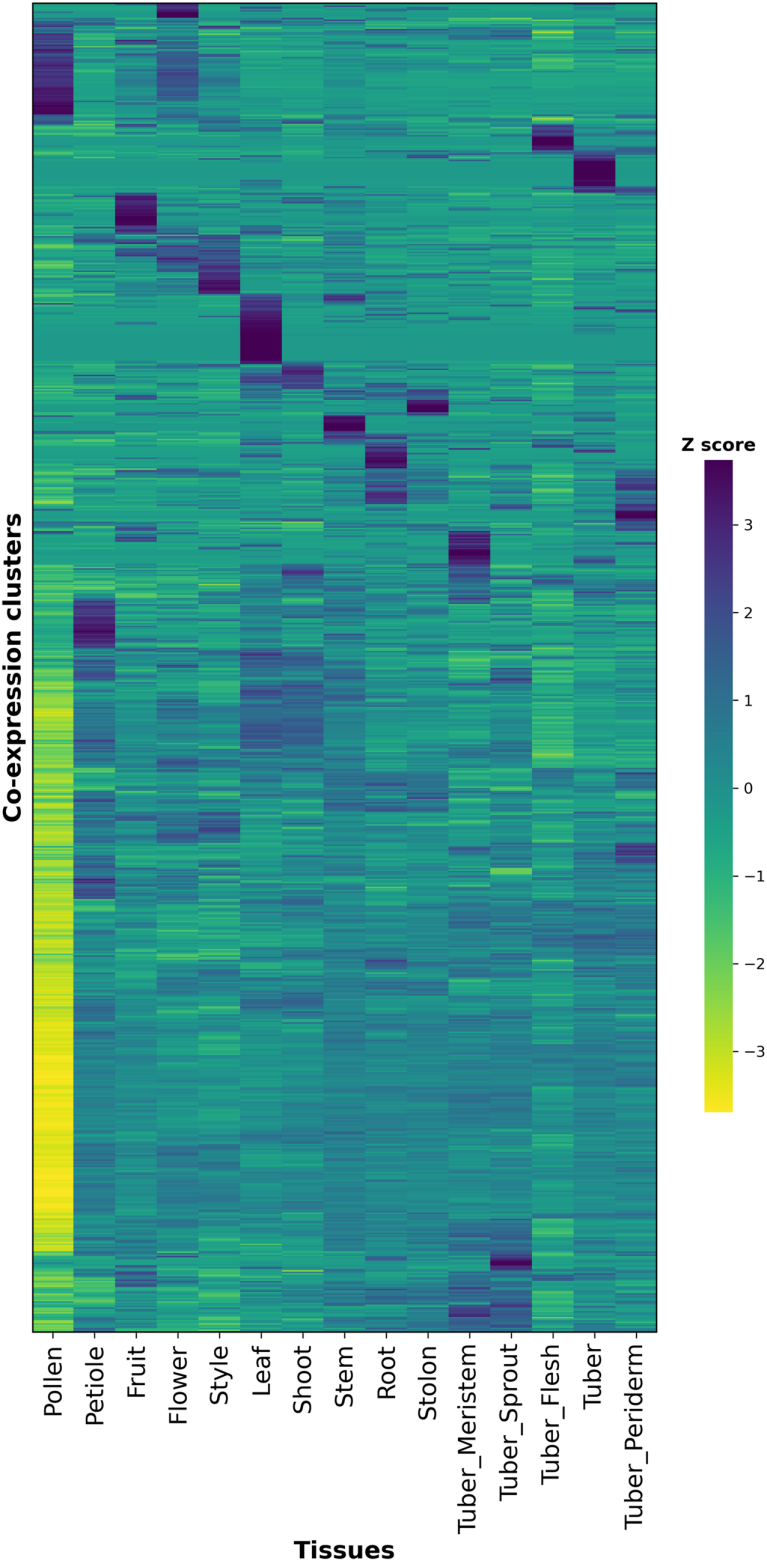
The clusters-tissues relationship heatmap. The heatmap shows that the tissues are on the x-axis, and the co-expression clusters are on the y-axis. The heatmap was colour-coded with a Z score of averaged expression level (TPM) per module at each tissue. Python was used to cluster the rows and columns hierarchically

To determine if our co-expression network has a scale-free architecture (Barabási and Bonabeau 2003), we calculated the Pearson correlation coefficient (PCC) for each pair of genes with a threshold of 0.5 and determined the number of times a particular gene is co-expressed with other genes at this threshold (node degree). We plotted the resulting power law distribution, which showed a negative correlation between node frequency (the number of genes with a certain number of connections) and node degree (the number of connections per gene). This distribution confirms the scale-free topology of our network (Figure S4). We constructed an HRR-based co-expression network using the above computed gene–gene PCC values using the CoNekT framework (Proost and Mutwil 2018). The constructed network contained 28,388 nodes representing genes and 4,57,580 edges representing associations between two nodes, such as HRR and PCC (Table S15). Using the heuristic cluster chiseling algorithm (Mutwil et al. 2010), we identified 853 clusters of co-expressed genes with the size of modules ranging from 2 to 285. We found that about 51% of co-expression clusters contained just two genes, while about 32% contained more than ten genes (Table S15). We visually assessed the quality of the identified clusters by inspecting the deviation of expression patterns of individual genes against the average expression pattern of the respective cluster. In this study, we considered genes with a Z score smaller than ± 1 as a tight co-expression in respective clusters. Based on these criteria, we found that an average of 85.39% of the genes across all clusters showed a tight co-expression (Figures S5 & S6). To understand the relationships between the identified clusters and tissues, we plotted a heatmap for the Z scores of the average expression level (TPM) per module at each tissue (Fig. 3), and we found about 54% (461) clusters that showed distinct expression patterns across tissue, i.e., Z score larger than ± 1 in at least three tissues. To understand the function of these clusters, we conducted an enrichment analysis, which revealed that more than 65% of the clusters contained at least one significantly enriched (corrected p-value < 0.05) biological process (Table S15). The identified clusters, thus, effectively grouped the genes that may participate in the same biological pathways and constitute the basis for identifying gene co-expression clusters underlying various agronomic traits.

### Co-expression clusters related to anthocyanin biosynthesis

**Fig. 4:**
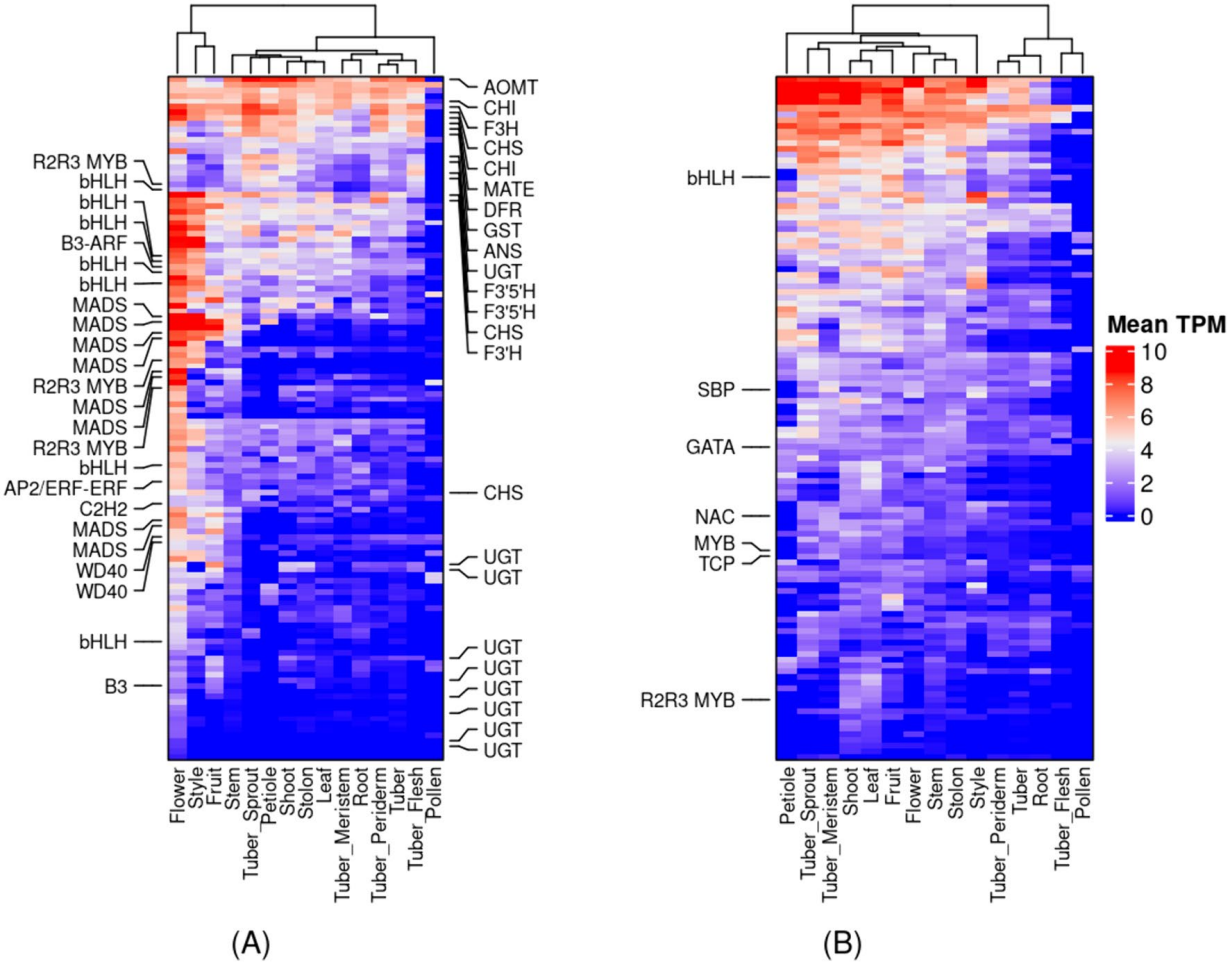
The heatmaps of co-expression clusters harboring genes that encode structural enzymes involved in the anthocyanin biosynthesis pathway in potatoes. **A** Cluster_90. **B** Cluster_78. The heatmaps show that the tissues are on the x-axis, and the genes are on the y-axis. The heatmaps were color-coded with log2 transformed mean TPM values for each tissue. We clustered the rows and columns hierarchically in Python. We marked the transcription factors on the left and the structural genes on the right side of the heatmap. The interactive clusters for Cluster_90 and Cluster_78 can be accessed at https://stcoexpnet.julius-kuehn.de/cluster/graph/434 and https://stcoexpnet.julius-kuehn.de/cluster/graph/439, respectively

We searched for co-expression modules associated with anthocyanin biosynthesis using the gene ontology (GO) term “anthocyanin-containing compound biosynthetic process (GO:0009718)” in our co-expression network. We found a single co-expression cluster, Cluster_90 (corrected *p*-value < 8.7e-05), containing genes including the ones that encode the structural enzymes involved in the anthocyanin biosynthesis (Table S16; Table S17), except the primary regulator gene, R2R3 MYB TF, the homolog of PhAN2 (R2R3 MYB TF), present on chromosome 10 (Jung et al. 2009). Nonetheless, in this cluster, we found three new MYB TFs (SOLTUB.AGRIA.G00000008919, SOLTUB.AGRIA.G00000017419, and SOLTUB.AGRIA.G00000019730) mapping to chromosomes 2 and 5. In addition, we also found 19 TFs belonging to bHLH, MADS, B3, C2H2 and AP2 TF families and two WD40 repeat-containing proteins in Cluster_90 (Fig. 4A; Table S18). Moreover, various GO terms such as “flavonoid biosynthetic process” (GO:0009813), “organic substance biosynthetic process” (GO:1,901,576), “pigment metabolic process” (GO:0042440), “DNA-binding transcription factor activity” (GO:0000981), and “anthocyanin-containing compound metabolic process” (GO:0046283) were significantly enriched (corrected *p*-value < 0.05) in this cluster (Table S19).

To find the primary regulatory gene of anthocyanin biosynthesis R2R3 MYB TF in our reference genome, we searched the reference protein sequences using the protein sequence of PhAN2 (UniProt ID: A4GRV2) as a query using BLASTP (Altschul et al. 1990). This search resulted in the identification of four homologs of PhAN2 in potatoes, mapping to chromosome 10. Three of them are present in three different clusters, namely Cluster_133, Cluster_78, and Cluster_85. In contrast, the fourth homolog does not have a cluster assignment. Among the three homologs, only one homolog (SOLTUB.AGRIA.G00000035098) is present in a cluster, Cluster_78, in which the GO terms “phenylpropanoid metabolic” (GO:0009698) and the “proanthocyanidin biosynthetic” (GO:0010023) processes were significantly enriched (corrected *p*-value < 0.05) (Table S20). Hence, we consider this gene as the homolog of PhAN2, which regulates the early biosynthetic genes in our reference genome (dAg). In addition, Cluster_78 also contains seven TFs belonging to MYB, TCP, NAC, SBP, GATA and bHLH TF families (Fig. 4B; Table S18; Table S21).

### Co-expression clusters related to tuberization

**Fig. 5:**
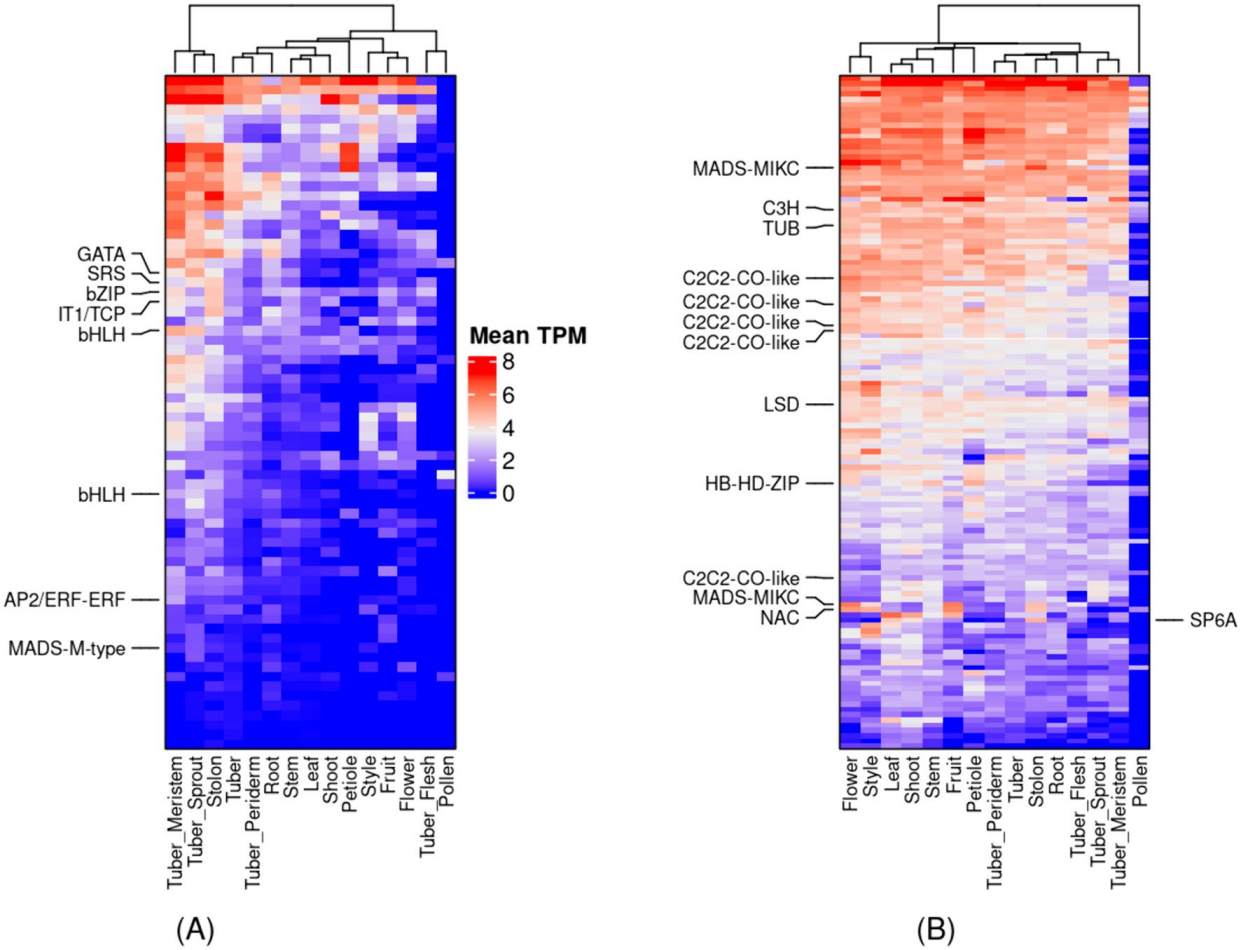
The heatmaps of co-expression clusters harboring tuberization genes, the tuber identity gene (IT1) and SELF-PRUNING 6A (SP6A). **A** Cluster_23. **B** Cluster_97. The heatmaps show that the tissues are on the x-axis, and the genes are on the y-axis. The heatmaps were color-coded with log2 transformed mean TPM values for each tissue. We clustered the rows and columns hierarchically in Python. We marked the transcription factors, including IT1, on the left side of the heatmap and the SP6A on the right side. The interactive clusters for Cluster_23 and Cluster_97 can be accessed at https://stcoexpnet.julius-kuehn.de/cluster/graph/473 and https://stcoexpnet.julius-kuehn.de/cluster/graph/689, respectively

We searched in our co-expression network for co-expression clusters harboring genes, StSP6A and IT1, involved in tuberization (Tang et al. 2022). We found two co-expression clusters, Cluster_23 and Cluster_97, containing IT1 and StSP6A, respectively. Cluster_23 contained seventy genes, including IT1 (Table S22; Fig. 5A). This cluster contained eight genes belonging to seven TF gene families: SRS, bZIP, bHLH, MADS-box, TCP, GATA, and AP2/ERF-ERF. These TFs are predominantly expressed in stolons, sprouting tubers or tuber meristem (Table S23). In addition, various GO terms such as “seed trichome elongation” (GO:0090378), “lipid transport” (GO:0006869), “the developmental process involved in reproduction” (GO:0003006), and “cellular process involved in reproduction in a multicellular organism” (GO:0022412) (Table S24) were significantly enriched (corrected *p*-value < 0.05) in this cluster. Cluster_97 contained 128 genes, including StSP6A (Table S25; Fig. 5B). This cluster contained 12 genes belonging to seven TF gene families, including C3H, TUB, LSD, MADS, C2C2-CO-like, HB-HD-ZIP, and NAC (Table S23). In addition, GO terms for hundreds of biological processes, including “regulation of long-day photoperiodism, flowering” (GO:0048586), “cellular response to light stimulus” (GO:0071482), “regulation of photoperiodism, flowering” (GO:2,000,028), “cellular response to radiation” (GO:0071478), and “response to red or far-red light” (GO:0009639), were significantly enriched (corrected *p*-value < 0.05) in this cluster (Table S26).

### Co-expression clusters related to defense responses

In this study, we identified 578 genes which belong to different classes of nucleotide-binding (NB) domain and leucine-rich repeat (LRR) (NLR) genes (Table S27) using NLR-Annotator (Steuernagel et al. 2020). A total of 432 out of 578 NLR genes were assigned to 119 co-expression clusters which contain 1–44 NLR genes per cluster. Among the 119 co-expression clusters, 43 were enriched for at least one biological process involved in defense mechanisms (Table S28), such as “response to biotic stimulus (GO:0009607)”, “defense response (GO:0006952)”, “response to fungus (GO:0009620)”, “defense response to fungus (GO:0050832)”, “response to bacterium (GO:0009617)”, “defense response to bacterium (GO:0042742)”, “response to virus (GO:0009615)”, and “defense response to virus (GO:0051607)”.

**Fig. 6:**
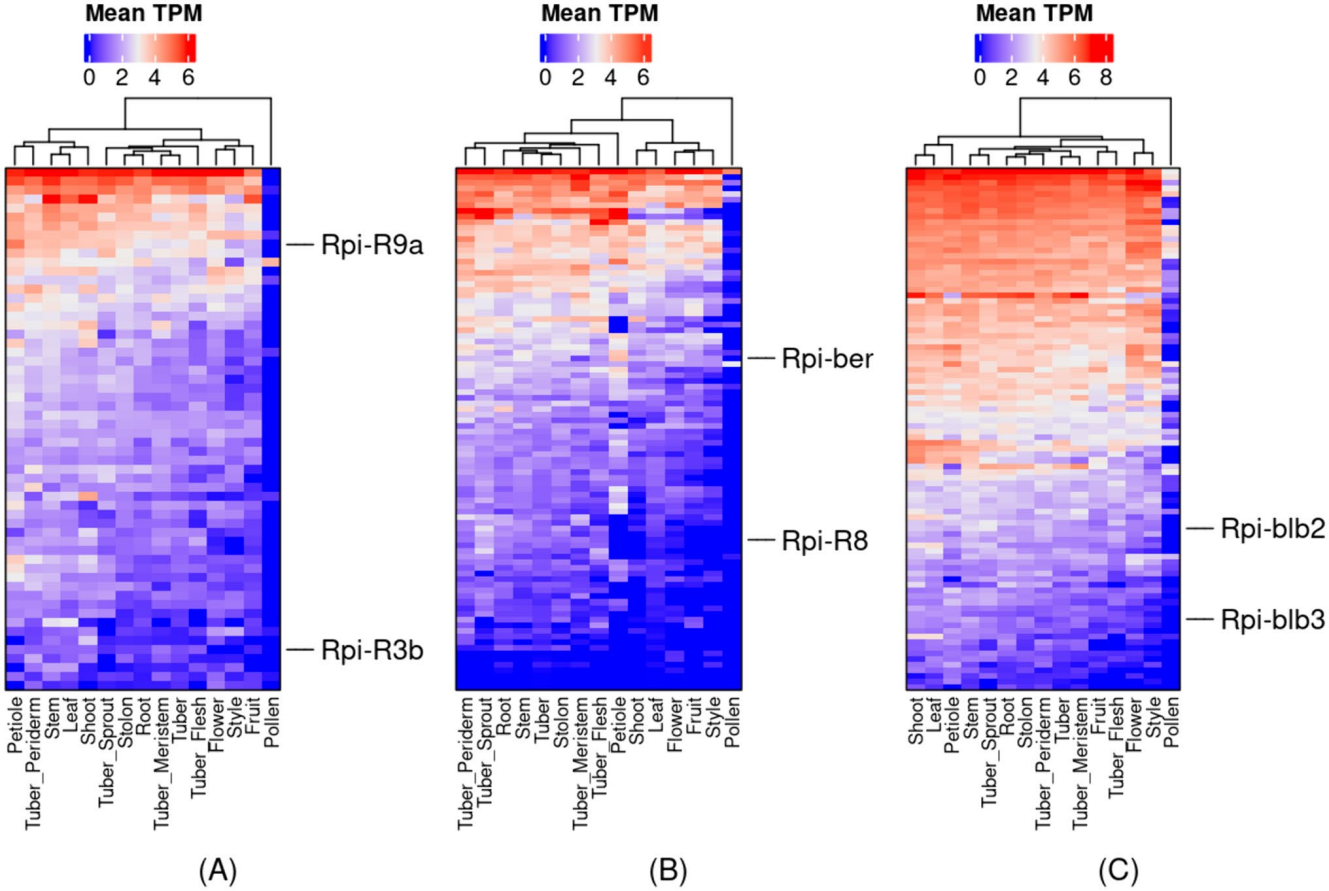
The heatmaps of co-expression clusters harboring genes involved in resistance against late blight disease. **A** Cluster_223; **B** Cluster_103; **C** Cluster_210. The heatmaps show that the tissues are on the x-axis, and the genes are on the y-axis. The heatmaps were color-coded with log2 transformed mean TPM values for each tissue. We clustered the rows and columns hierarchically in Python. We marked the identified NLRs effective against *Phytophthora infestans* (Rpi genes) on the right side of the heatmap. Rpi-vnt1.1 and Rpi-vnt1.1-A2056 are homologs to Rpi-R9a. The interactive clusters for Cluster_223, Cluster_103, and Cluster_210 can be accessed at https://stcoexpnet.julius-kuehn.de/cluster/graph/327, https://stcoexpnet.julius-kuehn.de/cluster/graph/479, and https://stcoexpnet.julius-kuehn.de/cluster/graph/322, respectively

We found eight of 14 known NLRs effective against *Phytophthora infestans* (Rpi genes) (Armstrong et al. 2019) in three co-expression clusters, Cluster_223, Cluster_210, and Cluster_103, while the remaining Rpi genes were either not assigned to any cluster or clusters did not enrich for any of the above-mentioned biological processes (Table S29). Cluster_223 contains 58 genes, of which 32 encode NLRs (Table S30; Fig. 6A). In this cluster, we found four Rpi genes, Rpi-R3b, Rpi-R9a, Rpi-vnt1.1, and Rpi-vnt1.1 A2056, mapping to two homologs in the reference genome, SOLTUB.AGRIA.G00000038927 and SOLTUB.AGRIA.G00000032822. In this cluster, the biological process “defense responses” to fungi, bacteria and viruses were enriched (Table S31). Cluster_103 contains 92 genes, of which 44 encode NLRs (Table S32; Fig. 6B). In this cluster, we found two Rpi genes, Rpi-R8 and Rpi-ber, mapping to two homologs in the reference genome, SOLTUB.AGRIA.G00000032965 and SOLTUB.AGRIA.G00000035214. In this cluster, the biological process “defense responses” to fungi and bacteria were enriched (Table S33). Cluster_210 contains 92 genes, of which 11 encode NLRs (Table S34; Fig. 6C). Similarly, this cluster contained two Rpi genes, Rpi-blb2 and Rpi-blb3, mapping to two homologs in the reference genome, SOLTUB.AGRIA.G00000044086 and SOLTUB.AGRIA.G00000013669. In this cluster, defense responses related to a specific pathogen were not enriched, but “response to biotic stimulus” and “defense response” were enriched (Table S35).

### Co-expression cluster related to self-incompatibility

**Fig. 7:**
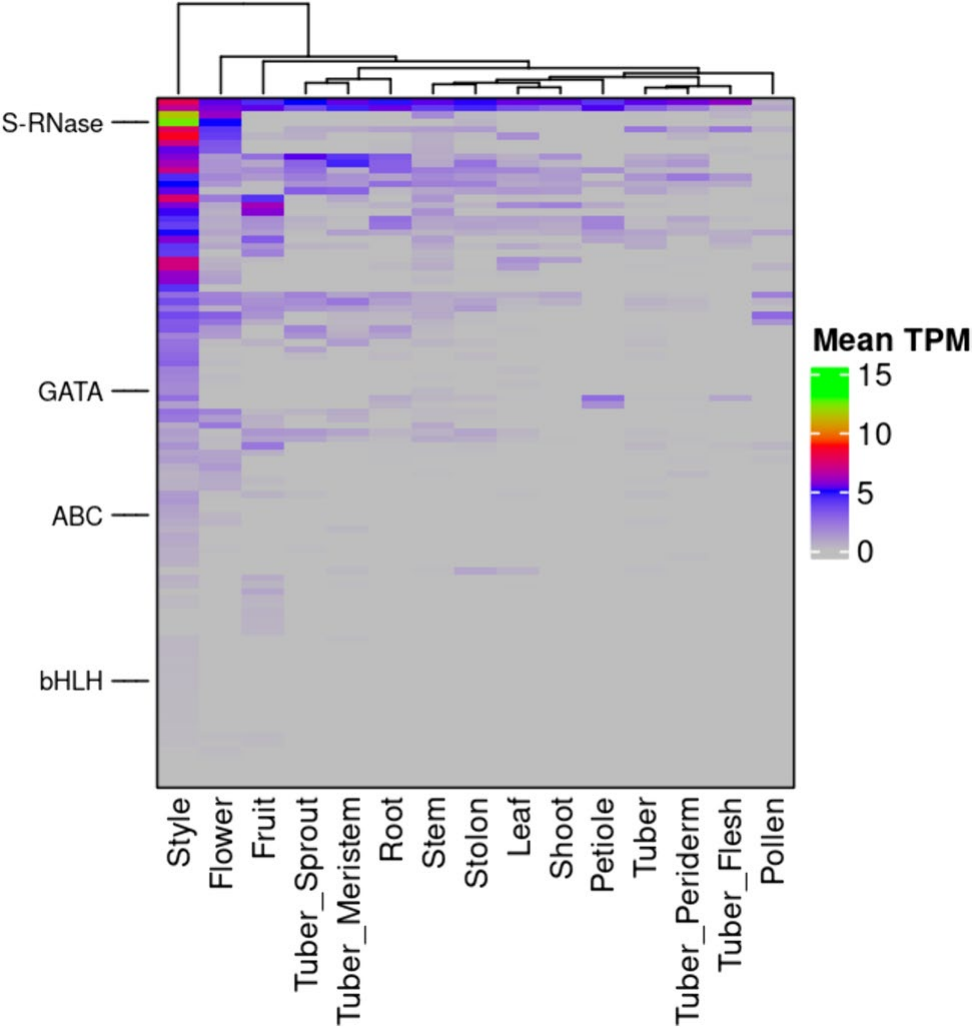
The heatmap of Cluster_30 that harbors the S-RNase gene. The heatmap shows that the tissues are on the x-axis, and the genes are on the y-axis. The heatmap was color-coded with log2 transformed mean TPM values for each tissue. We clustered the rows and columns hierarchically in Python. We marked this cluster's essential genes, S-RNase, ABC transporter (ABC), and two transcription factors (bHLH and GATA). The interactive cluster for Cluster_30 can be accessed at https://stcoexpnet.julius-kuehn.de/cluster/graph/367

We searched in our co-expression network for clusters harboring genes involved in self-incompatibility (Dzidzienyo et al. 2016). We found one co-expression cluster, Cluster_30, containing the S-RNase gene SOLTUB.AGRIA.G00000001844 which showed an extreme expression in style samples (mean TPM of 5783.82). This cluster contained 99 more genes and the majority of these genes showed high mean expression in style samples (Table S36; Fig. 7). However, surprisingly, we found no enriched GO terms in this cluster. Furthermore, this cluster contained two genes belonging to two TF gene families: GATA and bHLH.

### Data availability through a web server

The data presented above are easily accessible by researchers to explore the expression atlas of 2299 transcriptome samples and the co-expression network interactively via a web server called StCoExpNet. This web server is freely available at https://stcoexpnet.julius-kuehn.de.

## Discussion

### High-quality of publicly available potato RNA-Seq data

Publicly available RNA-Seq datasets, such as at NCBI SRA database, provide a wealth of information that can be used to investigate gene expression, alternative splicing, identify novel transcripts and identify functionally related genes in an organism. Researchers can use these datasets to test hypotheses, validate findings, and generate new insights into the mechanisms of various biological processes (Ferrari and Mutwil 2020; Wisecaver et al. 2017; Lin et al. 2017; Ramšak et al. 2018). In this study, we have performed extensive literature mining and constructed a global gene expression atlas for potatoes using thousands of publicly available RNA-Seq datasets (Figure S1). Our analyses revealed that these datasets clustered according to transcript abundance into three broad categories of tissues: pollen, aerial and underground tissues. Less than 2% of the analyzed RNA-Seq samples were excluded based on clustering analyses, indicating a high-quality level of the publicly available samples supported by the sample clustering in this study (Fig. 1). However, potato is highly heterozygous and in most cases tetraploid. When mapping reads from such samples onto a single haploid reference genome (dAg), collapsing multiple alleles into one is expected but it will not negatively influence the result of our study, as we are aiming to make in our study conclusions regarding the expression of genes and not individual alleles.

### New internal reference genes for qPCR experiments in potatoes

Housekeeping (HK) genes are those expressed constitutively across broad conditions and robustly (Czechowski et al. 2005; Bustin et al. 2009) and are used as internal reference genes in real-time quantitative polymerase chain reaction (qPCR) assays (Nicot et al. 2005; Hu et al. 2009; Tang et al. 2017). By utilizing the extensive compilation of RNA-Seq datasets presented in this study, one can assess the suitability of commonly utilized internal reference genes and put forward novel ones.

We identified 281 HK genes (Table S6) that showed a stable expression (Fig. 2A & B) across samples, supporting their suitability for use as internal reference genes in potato qPCR assays. The list of 281 HK genes includes three known reference genes, namely Elongation factor 1-alpha, 60 s ribosomal protein L8 **(**Nicot et al. 2005; Tang et al. 2017**) **and Ubiquitin-associated/translation elongation factor EF1B protein **(**Mariot et al. 2015**)**, used as internal reference genes in qPCR potato experiments under a few different stress conditions. Because these three HK genes exhibit a consistently stable expression across thousands of samples generated under various experimental conditions, these are particularly recommended to be used as internal reference genes for qPCR assays of potatoes (Table 1). Further, we found homologs of known reference genes of other crops in our list of potato HK genes. For example, Heat shock protein 90 was validated as a reference gene in *Cajanus cajan* under heat and salt stress conditions (Sinha et al. 2015). Eukaryotic initiation factor 4A was validated as a reference gene in *Carica papaya* under different experimental conditions (Zhu et al. 2012). YT521-B-like protein family protein was validated as a reference gene in perennial ryegrass (Lee et al. 2010), and Ubiquitin-conjugating enzyme E2 was validated as a suitable reference gene for *Eucommia ulmoides* Oliv under different experimental conditions (Ye et al. 2018a, b). Therefore, given the high expression stability of these four HK genes across many samples generated under different experimental conditions (Table 1), these genes could be considered as novel reference genes for potato qPCR experiments.

**Table 1:** List of known and novel internal reference genes used in potato qPCR assays identified in this study

Reference gene ID	Gene name	Mean TPM	SD	Tau index	References
SOLTUB.AGRIA.G00000040002*	60 s ribosomal protein l8 (L8)	8.926	0.996	0.0805	Tang et al. (2017)
SOLTUB.AGRIA.G00000020781*	Elongation factor 1-alpha (EF-1-alpha)	9.745	1.18	0.0838	Tang et al. (2017)
SOLTUB.AGRIA.G00000022993*	Ubiquitin-associated/translation elongation factor EF1B protein (C2)	5.303	0.552	0.1435	Mariot et al. (2015)
SOLTUB.AGRIA.G00000027916	Eukaryotic initiation factor 4A (EIF)	7.489	0.743	0.0816	Zhu et al. (2012)
SOLTUB.AGRIA.G00000026336	Heat shock protein 90 (HSP90)	10.909	1.319	0.0899	Sinha et al. (2015)
SOLTUB.AGRIA.G00000001694	ARATH YTH domain-containing protein ECT2	8.313	1.114	0.1246	Lee et al. (2010)
SOLTUB.AGRIA.G00000011879	Ubiquitin-conjugating enzyme E2	7.625	0.806	0.1065	Ye et al. (2018a, b)

Mean TPM, SD and Tau indexes indicate the mean TPM across all RNA-Seq samples, standard deviation, and tissue-specificity index, respectively

^*^Indicates known reference genes

### The global co-expression network and co-expression clusters

Scale-free networks follow a power-law distribution, where a few genes are highly connected while most genes have only a few connections (Barabási and Bonabeau 2003). This structure was believed to be an evolved feature that ensures stability and robustness against genetic and environmental disturbances (Barabási and Oltvai 2004). We found that the potato’s co-expression network follows this scale-free topology and supports the biological validity of our expression data (Figure S4). Most of the genes found in all clusters displayed a tight co-expression that suggests the genes in respective modules have similar expression patterns (Figure S5 and S6), indicating a high quality of the identified clusters. Further, we found several clusters positively related to specific tissues that may suggest that the genes within the co-expression cluster are actively involved in biological processes specific to that tissue. In contrast, many clusters are negatively related to pollens (Fig. 3).

Clusters of functionally related genes tend to have strong connections within the co-expression network. Identifying and examining these clusters can help to uncover the functional gene clusters of an organism (Mutwil et al. 2010; Rhee and Mutwil 2014; Aoki et al. 2016). In order to illustrate that the identified co-expression clusters are biologically interpretable, we discuss as proof of concept the ones related to important potato agronomic traits, including anthocyanin biosynthesis, tuberization, defense responses against multiple pathogens, and self-incompatibility.

### Transcriptional complexity of anthocyanin production in potatoes

Anthocyanins are plant secondary metabolites that are responsible for the vibrant coloration of various plant tissues (Laimbeer et al. 2020). They have gained significant attention due to their numerous documented benefits for plants’ physiological processes and human health (Stintzing and Carle 2004; De Pascual-Teresa and Sanchez-Ballesta 2008; Khoo et al. 2017; Schulz et al. 2016; Merzlyak and Chivkunova 2000). In solanaceous species, the early biosynthetic genes (EBGs), which include chalcone synthase (CHS), chalcone isomerase (CHI), flavonoid 3-hydroxylase (F3H), and flavonoid 3′ hydroxylase (F3′H) are regulated by the R2R3 MYB transcription factor (TF) (Jung et al. 2009). The late biosynthetic genes (LBGs), which include flavonoid 3′-5′ hydroxylase (F3′5'H), dihydroflavonol 4-reductase (DFR), anthocyanidin synthase (ANS), glutathione S-transferase (GST), anthocyanin O-methyltransferase (AOMT), and glucosyl transferases (UFGT), are regulated by a ternary protein complex called MBW in a spatiotemporal manner. The complex is formed from MYB, basic helix loop helix (bHLH) TFs, and WD40 repeat-containing proteins (Patra et al. 2013; Lin-Wang et al. 2010; Feller et al. 2011). Finally, the synthesized anthocyanins will be transported to the vacuole by the MATE transporter (Gomez et al. 2009). The genetic basis of the anthocyanin biosynthetic pathway has been studied in potatoes and identified several essential genes involved in this pathway (Jung et al. 2009; Zhang et al. 2009a; Zhang et al. 2009b; Śliwka et al. 2017; Laimbeer et al. 2020). However, so far, only a few studies have been conducted to investigate the transcriptional dynamics of the identified vital genes between different colored phenotypes (Laimbeer et al. 2020; Riveros-Loaiza et al. 2022). Moreover, these studies were conducted using single tissue of a small number of clones. Hence, limited information on the global transcriptional complexity of anthocyanin biosynthesis in potatoes is available. Therefore, we investigated the transcriptional complexity of anthocyanin production in potatoes using a global co-expression network in this study.

We found a single co-expression cluster (Cluster_90) that contains 24 TFs and 23 genes that encode various structural enzymes involved in the anthocyanin biosynthetic pathway (Fig. 4A; Tables S17 & S19). Hence, we associated this cluster with anthocyanin biosynthesis in potatoes. The sum of the TFs and genes belonging to Cluster_90 is more than double the number of genes identified in a recent study conducted to investigate the transcriptional dynamics between genotypes with different colorations of flesh and skin (Riveros-Loaiza et al. 2022), illustrating that the global co-expression network approach is robust and efficient in identifying genes underlying agronomic traits. The newly identified TFs may play an essential role in anthocyanin biosynthesis in potatoes. For example, we identified three TFs belonging to the MYB TF family in this cluster, mapped to others than chromosome 10 (Jung et al. 2009). In addition, these genes showed above-average expression in multiple tissues (Figure S7). Therefore, we hypothesize that several homologs of PhAN2 (R2R3 MYB TF) may transcriptionally regulate the anthocyanin biosynthesis in different tissues spatiotemporally in potatoes. Further, we identified eight TFs belonging to the MADS-box TF family in this cluster. A SQUAMOSA-class MADS-box TF, VmTDR4, is associated with anthocyanin biosynthesis during normal ripening in bilberry (Jaakola et al. 2010). Hence, these MADS-box TFs may also play an essential role in potato anthocyanin biosynthesis. This cluster provided several genes that may help define future breeding strategies to develop new potato cultivars with high anthocyanin content.

Further, we identified the primary regulator of anthocyanin biosynthesis, R2R3 MYB TF, in a different cluster, Cluster_78. Cluster_78 contains several TFs (Fig. 4B; Tables S20 & S21) and many genes involved in the phenylpropanoid metabolic process, providing precursors for anthocyanin biosynthesis (Laimbeer et al. 2020). Thus, this cluster can be associated with the phenylpropanoid metabolic process and anthocyanin biosynthesis and illustrates the mechanistic interlink between both pathways which was not previously reported in potatoes.

### Transcriptional complexity of tuberization in potatoes

In general, late-maturing cultivars (LMC) produce higher yields than early-maturing cultivars (EMC). However, abiotic stresses, such as heat waves and drought, negatively affect the tuber quality and yields of LMCs. In contrast, EMCs escape these stress conditions. The early induction of tuberization dictates the time to crop maturity and is an essential agronomic trait that lies in its ability to influence the overall yield over an extended period. On the molecular, it is known that leaves act as sensors for day length and generate a mobile signal known as tuberigen, which is then transported to the underground stems to trigger the process of tuberization (Zierer et al. 2021). The FLOWERING LOCUS T (FT) protein (StSP6A) controls potato tuberization (Navarro et al. 2011). In addition, a TCP TF, called *Identity of Tuber 1* (*IT1*), interacts with StSP6A and forms a protein complex which regulates the tuber initiation (Tang et al. 2022). Nevertheless, insights into the transcriptional complexity behind tuberization still need to be discovered that may identify unknown genes playing an essential role in tuber development. Therefore, we investigated the transcriptional complexity of potato tuberization using a global co-expression network in this study.

In this study, we found two co-expression clusters based on the presence of the two essential genes involved in the regulation of potato tuberization, IT1 (Cluster_23; Fig. 5A) and StSP6A (Cluster_97; Fig. 5B). In addition, these two clusters enriched significantly for biological processes involved in the photoperiodic control of tuberization, day-length dependent tuberization, response to light stimulus, elongation of stolons, and transporting biomolecules (Table S24 & Table S26). Therefore, we associated these two clusters with the regulation of tuberization in potatoes. We found multiple TFs, such as bZIP, CO, and TCP, that are known to play an essential role in regulating tuberization by forming the tuberigen activation complex (TAC) (Teo et al. 2017) and other complexes similar to TAC (Tang et al. 2022). In addition, we found new TFs in these two clusters belonging to multiple TF families, such as C3H, TUB, LSD, NAC, SRS, bHLH, GATA, MADS-box, HB-HD-ZIP and AP2, and these TF may be directly or indirectly involved in the regulation of tuberization (Table S23; Fig. 5A & B). For example, researchers have discovered a MADS-box TF (IbSRD1) in sweet potatoes that responds to auxin and promotes the proliferation of metaxylem and cambium cells. The overexpression of IbSRD1 led to earlier thickening of storage roots, indicating that the gene is involved in regulating the initial growth of storage roots in an auxin-dependent manner (Noh et al. 2010). Therefore, the newly identified TFs provide us with new targets in breeding programs to improve the earliness of varieties and, thus, escape adverse abiotic stress conditions.

### Transcriptional complexity of defense responses against multiple pathogens

Plants possess cell surface and intracellular receptors, which can detect molecules produced by pathogens and trigger defense responses. The nucleotide-binding (NB) domain and a leucine-rich repeat (NLR) genes are important but not the only defense responsive genes. All these genes accomplish the defense responses by detecting the molecules secreted by pathogens and activating a suite of dense response processes against the pathogens (Feehan et al. 2020). In this study, we identified 578 NLR genes (Table S27), which is significantly lower than the number of predicted NLR genes for most potato accessions (Tang et al. 2022) but slightly higher than for the wild relatives of sweet potato species, *Ipomoea trifida* (547 NLR genes) and *Ipomoea triloba* (569 NLR genes) (Wu et al. 2018). We found 226 NLR genes present in 43 co-expression clusters, which enriched for various biological processes involved in defense responses, of which several co-expression clusters enriched for defense response processes against multiple pathogens (Fungi/Bacterium/Virus) (Table S28).

*Phytophthora infestans* is the major pathogen in potato and causes late blight disease. Several functional NLR genes effective against *Phytophthora infestans* (Rpi genes) have been successfully cloned (Armstrong et al. 2019; Paluchowska et al. 2022). Several transcriptomic studies have been conducted to identify differentially expressed genes between contrasting potato cultivars for late blight disease (Duan et al. 2020; Cao et al. 2020; Yang et al. 2018). However, the transcriptional regulation of these Rpi genes remains unknown. In this study, we identified eight of 14 NLRs that have been reported as effective against *Phytophthora infestans* (Rpi genes) in three co-expression clusters (Cluster_223, Cluster_103, and Cluster_210) along with 79 other NLR genes and five TFs belonging to EIL, C3H, C2H2, NAC and MYB TF families (Tables S30, S32 and S34; Fig. 6A, B and C).

The identified TFs may regulate the Rpi genes directly or indirectly to confer resistance against the pathogens. For example, an MYB TF increases resistance against the pathogen, *Botryosphaeria dothidea* in apples by regulating circular wax biosynthesis (Zhang et al. 2019). In addition, numerous studies have investigated the role of NAC transcription factors in plant immunity and identified dozens of NAC genes that function as positive or negative regulators of plant immunity, as well as modulators of hypersensitive response and stomatal immunity, or targets of pathogen effectors (Yuan et al. 2019). Furthermore, a novel protein elicitor (SsCut) from *Sclerotinia sclerotiorum* induces multiple defense responses in plants, Arabidopsis, soybean, rice, maize and wheat by causing hypersensitive response (HR). In addition, SsCut increases plant resistance to multiple pathogens, *S. sclerotiorum*, *Phytophthora nicotianae* and *Phytophthora sojae*. A Virus-induced gene silencing revealed that C2H2 TF acts as a regulator of SsCut-triggered immunity in *Nicotiana benthamiana* (Zhang et al. 2014, 2016b).

The newly identified TFs and the above-described NLR genes could be targeted in the breeding program to develop new potato cultivars with resistance to multiple pathogens, especially late blight disease.

### Novel candidate genes to overcome self-incompatibility

Transforming the clonal crop potato into a diploid inbred/F1 hybrid variety presents an opportunity to employ efficient breeding techniques (Lindhout et al. 2011). Inbred potatoes could expedite the development of novel varieties with desired combinations of alleles for increased yield, tuber quality, and resistance traits (Jansky et al. 2016). However, a major obstacle to this strategy is the prevalence of gametophytic self-incompatibility (SI) in most diploid potato germplasm, hindering the creation of diploid homozygous lines. SI is a reproductive isolation mechanism observed in plant species of about 60 plant families, including Solanaceae. In the Solanaceae, the style distinguishes between self and non-self-pollen to inhibit self-fertilization and promote outcrossing (Dzidzienyo et al. 2016). A single polymorphic locus, called the S-locus, governs the SI in potato (Fujii et al. 2016). This locus encompasses two distinct determinants: the female/pistil S-determinant, which is a cytotoxic S-ribonuclease known as S-RNase, and the male/pollen S-determinant, which consists of a group of pollen-specific S-locus F-box proteins called SLFs (McClure et al. 1989; Ushijima et al. 2003). The S-RNase functions by impeding the growth of self-pollen tubes through either ribosomal RNA (rRNA) degradation or disruption of the cytoskeleton’s dynamic equilibrium (McClure et al. 1990; Roldán et al. 2012). During cross-pollination, based on the collaborative non-self-recognition system, the pollen-expressed SLFs recognize S-RNases and target them to the proteasomal degradation pathway, allowing pollen tube growth towards the ovaries where fertilization can take place (Kubo et al. 2010). On the other hand, the S-RNase is not degraded during self-pollination as it was not recognized by the self-SLFs that induce SI (Kubo et al. 2015).

Currently, two approaches are available to overcome the SI in potatoes and have been reported to confer self-compatibility (SC). (i) manipulation of S-RNase (Ye et al. 2018b; Enciso-Rodriguez et al. 2019)**. **Although this method converted SI genotypes to SC, the SC mutant lines produced varying numbers of seeds per fruit (67–288) across mutant lines, raising concerns about the method's robustness. (ii) introgression of the S-locus inhibitor (Sli) gene from wild potatoes into commercial varieties through conventional breeding (Hosaka and Hanneman 1998; Birhman and Hosaka 2000). This method is both time-consuming and demanding in terms of labor. In addition, this method relies on introgressing an allele from a wild species characterized by extended stolons and elevated levels of toxic steroidal glycoalkaloids in tubers in cultivated potato (Leisner et al. 2018). Moreover, these approaches are S-RNase-centric and aim to inhibit the functions of S-RNase to solve SI in potatoes. Hence, we advocate redirecting attention away from S-RNase and towards other candidate genes implicated in potatoes' self-incompatibility (SI) mechanism. Consequently, it is imperative to devise effective methodologies centered on these alternative candidate genes and utilize genotypes possessing the desired traits.

In this study, we found a co-expression cluster (Cluster_30) comprising the S-RNase gene, and hence, we associated this cluster with SI (Table S36). The majority of the genes in this cluster showed a high average expression in style tissue samples, suggesting the role of these genes in SI or biological processes related to SI (Fig. 7). Further, we analyzed this S-RNase gene’s immediate neighborhood in the co-expression network (Fig. 7). We found a member of the ABC transporter family (SOLTUB.AGRIA.G00000013767), to be co-expressed with the S-RNase. This ABC transporter may be potentially involved in transporting the S-RNase from pistil to pollen to accomplish SI in potatoes similar to what was reported for apples (Meng et al. 2014). Therefore, we hypothesize that disrupting the ABC transporter gene’s function by introducing mutations (Ye et al. 2018b; Enciso-Rodriguez et al. 2019) may block S-RNase transport to pollen from the pistil, leading to inducing SC.

### Data availability through a web server

A web server StCoExpNet has been created for researchers to explore the constructed potato expression atlas and gene co-expression network by adopting the CoNekT framework. This platform was chosen as it allows rich visualization features along with a detailed graphical user manual (Proost and Mutwil 2018). Our web server has the potential to serve as a reference database for potato transcriptomic studies. Through this resource, one can prioritize genes based on their expression and co-expression for mutagenesis, QTL cloning and GWAS studies. In addition, this resource can be used to investigate the gene expression and co-expression of the whole gene family of interest at the genome scale. Further, the results obtained from this resource can be mapped to different potato reference genomes through the integrated ortholog relationships among eight potato genotypes. Moreover, the expression atlas and the co-expression network can be downloaded through this web interface for local use. We are confident that this website will enhance data reuse and assist research groups in their projects.

## Conclusions

We have used an extensive collection of publicly available RNA-Seq datasets to construct a global transcriptome atlas for potatoes. We implemented a pipeline with state-of-the-art methods to map reads and quantity gene expression levels in 15 tissues. This atlas allowed us to identify housekeeping (HK) and tissue-specific (TS) genes. The HK genes might be used as internal reference genes in qPCR experiments, whereas TS genes might help researchers to test hypotheses in functional genomics studies. We also constructed a global gene co-expression network (GCN) for potatoes to explore the system-wide transcriptional landscape of potato tissues. We explored the functions of co-expression clusters using the gene ontology enrichment method. Several of the identified co-expression clusters are strongly linked with various agronomic traits. Our analyses revealed several candidate genes for various agronomic traits, and these can be used in defining future potato breeding programs. Furthermore, the present GCN sheds light on the functions of multiple potato genes and co-expression clusters. These findings are likely significant not only for understanding the roles of these genes but also for identifying genes that contribute to relevant agronomic characteristics. To enhance the reusability of the collected data, we developed a user-friendly web interface that enables the community to access and navigate through the data quickly. This resource will serve as a valuable asset not just for fundamental research endeavors but also for advancing innovative approaches aimed at boosting potato yield to meet the ever-growing global food requirements.

### Supplementary Information

Below is the link to the electronic supplementary material.

Supplementary file1 (PPTX 829 KB)

Supplementary file2 (XLSX 501 KB)

Supplementary file3 (XLSX 230 KB)

Supplementary file4 (XLSX 119 KB)

Supplementary file5 (XLSX 464 KB)

## Data Availability

The datasets analyzed during the current study are available in Supplementary Information. The interactive gene expression atlas and co-expression network are available at https://stcoexpnet.julius-kuehn.de.
