# Supplementary figures and images for: StCoExpNet: a global co-expression network analysis facilitates identifying genes underlying agronomic traits in potatoes
Source: Plant Cell Rep. 2024 Apr 15;43(5):117. doi: 10.1007/s00299-024-03201-2 (PMC11018665; doi:10.1007/s00299-024-03201-2)

## Slide 1
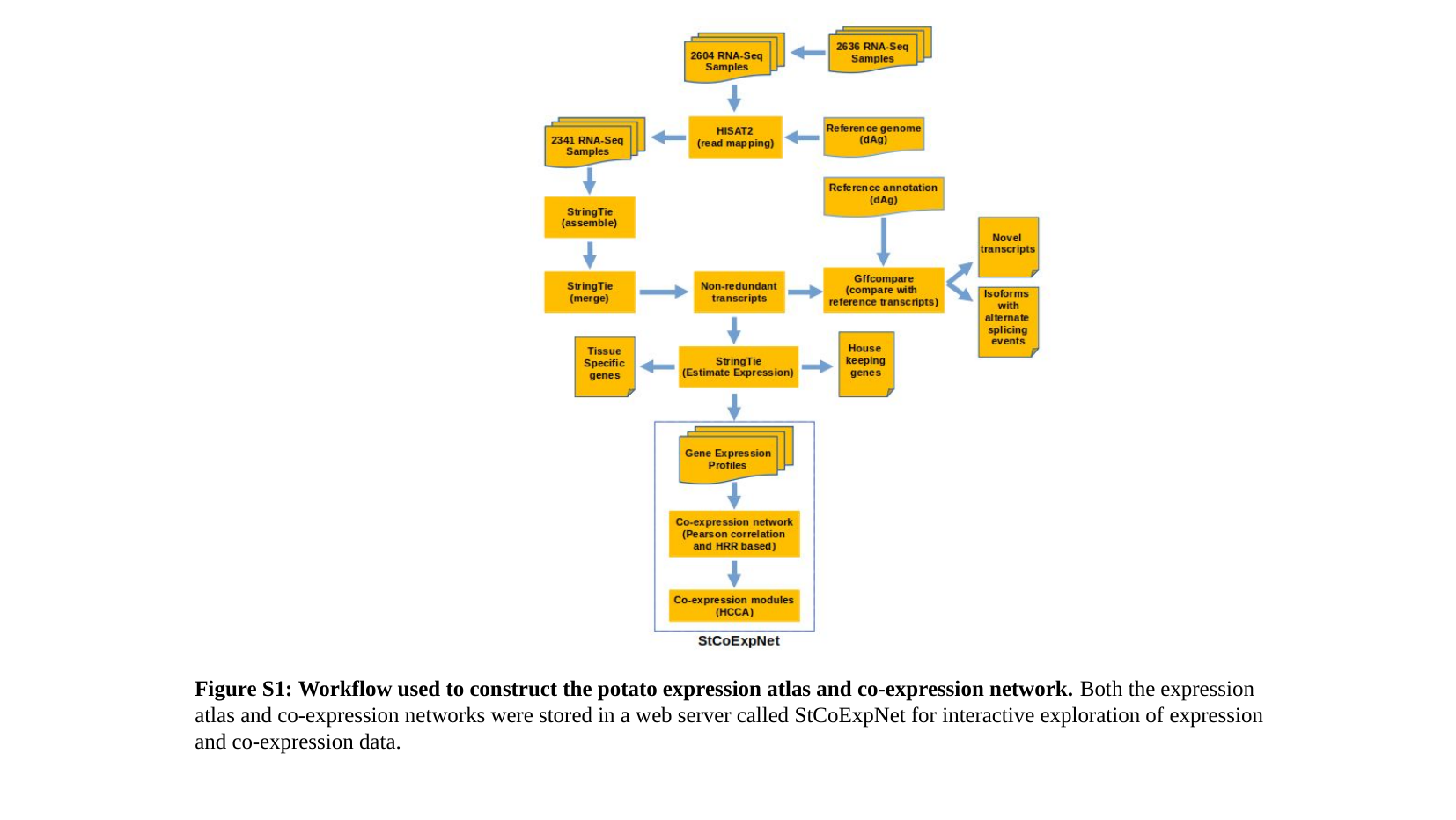

## Slide 2
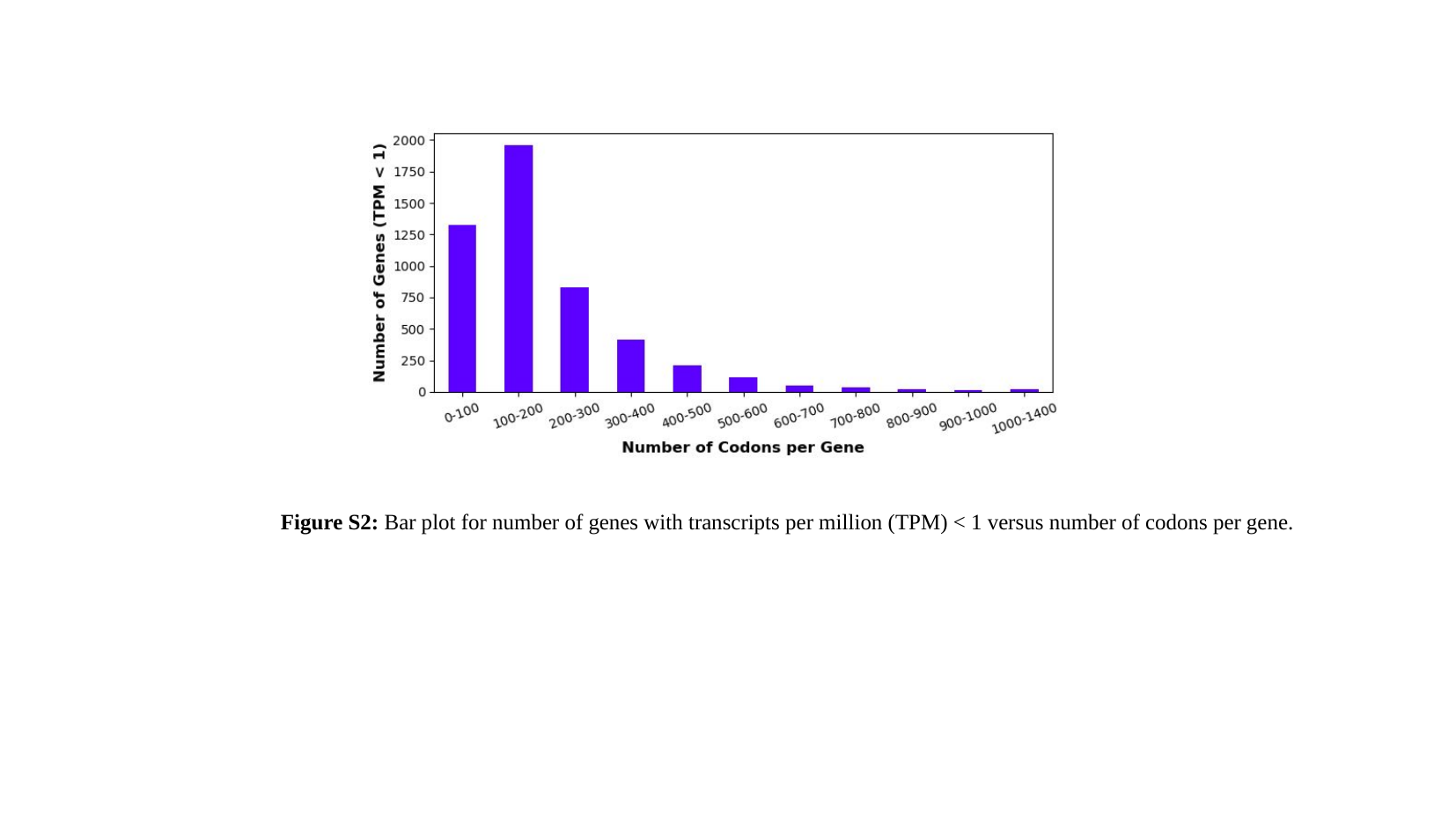

## Slide 3
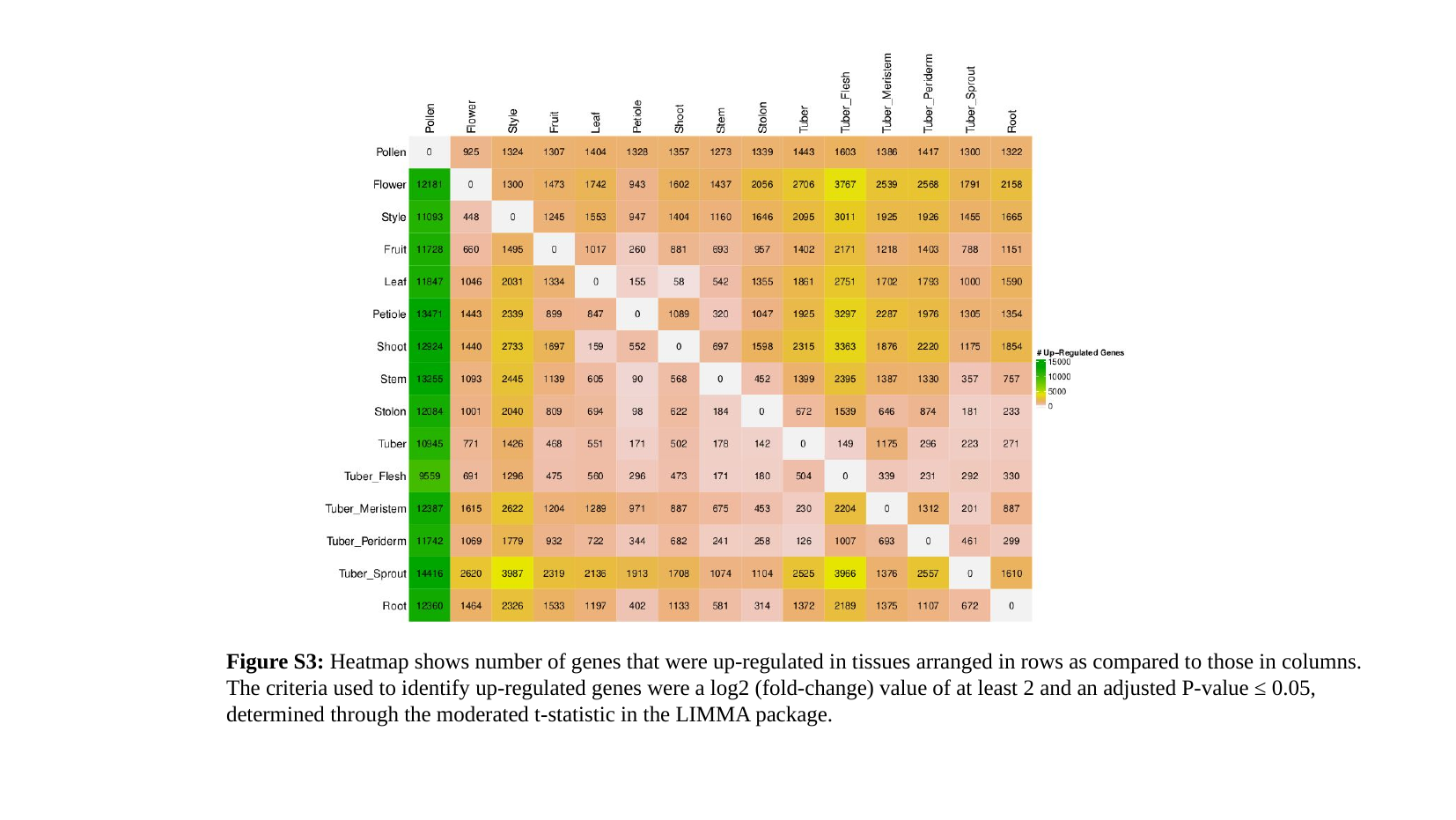

## Slide 4
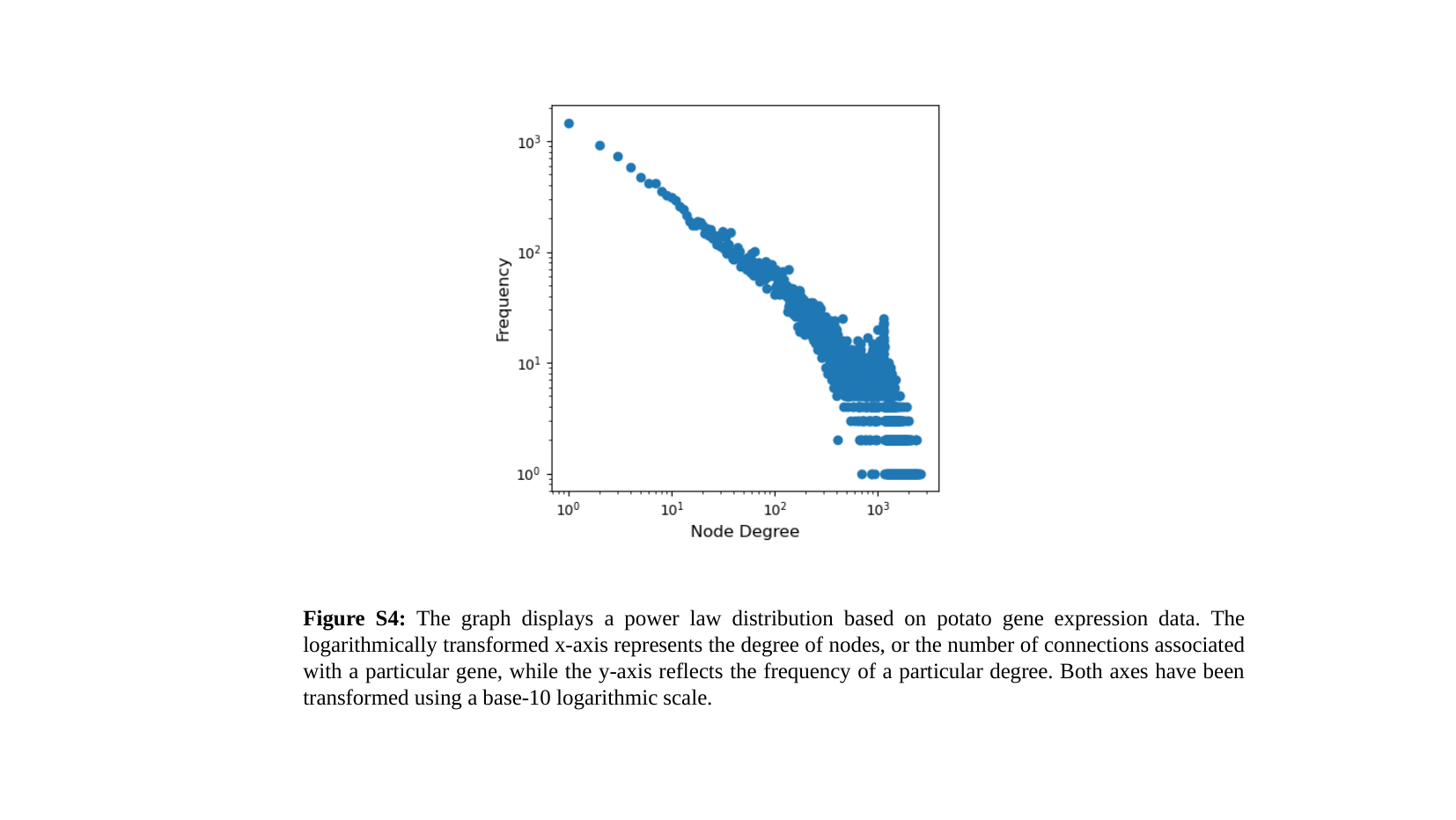

## Slide 5
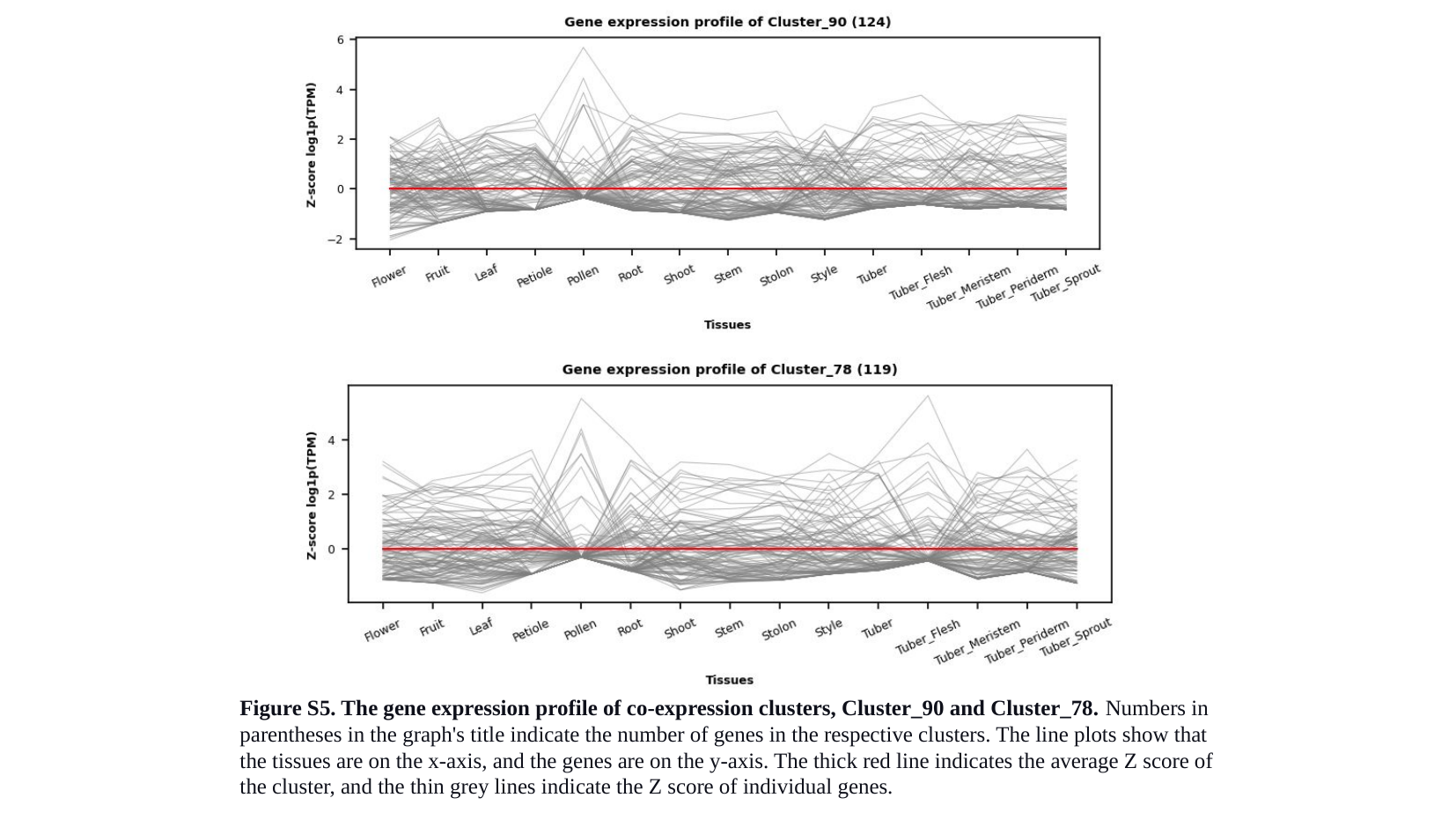

## Slide 6
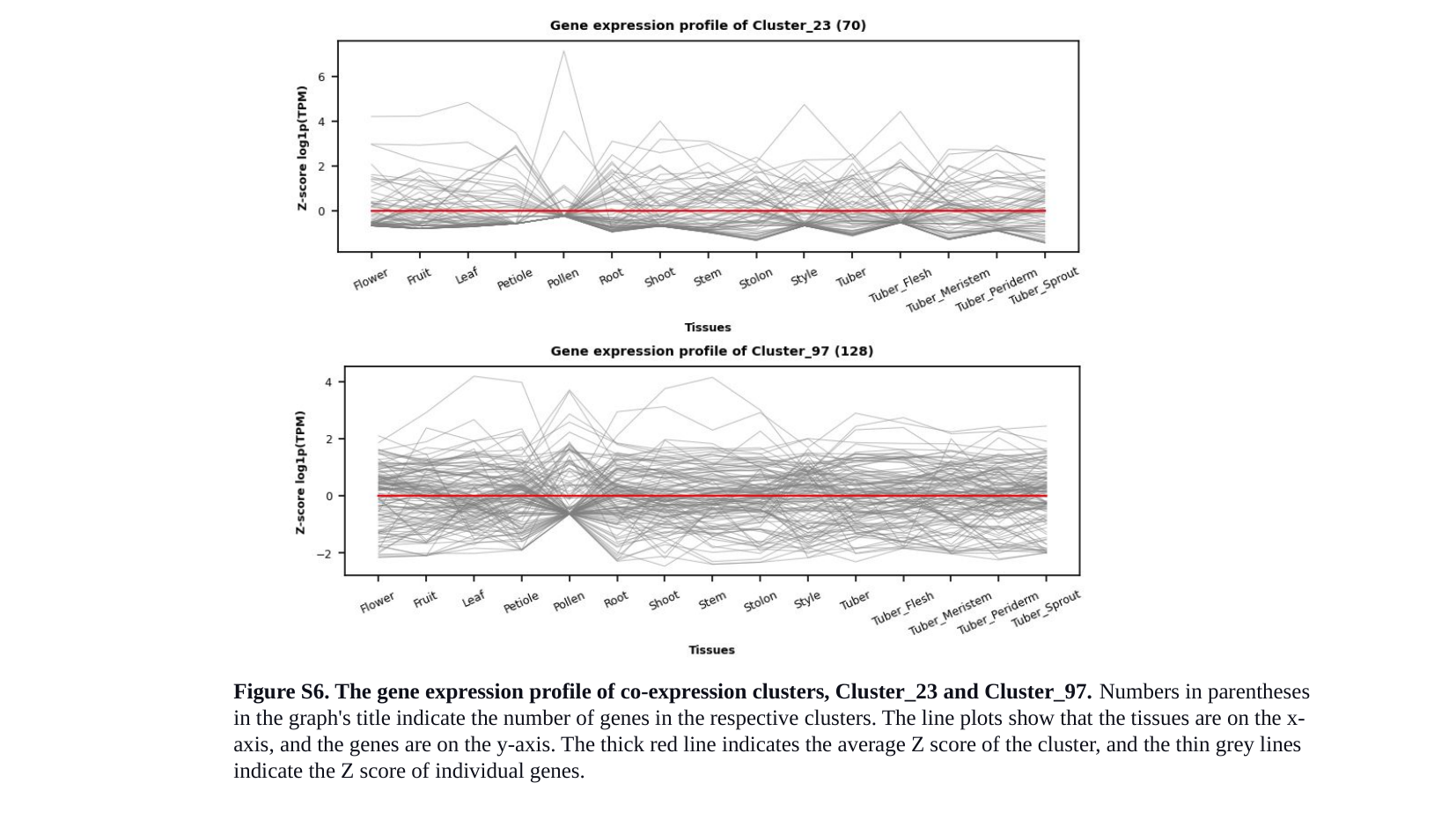

## Slide 7
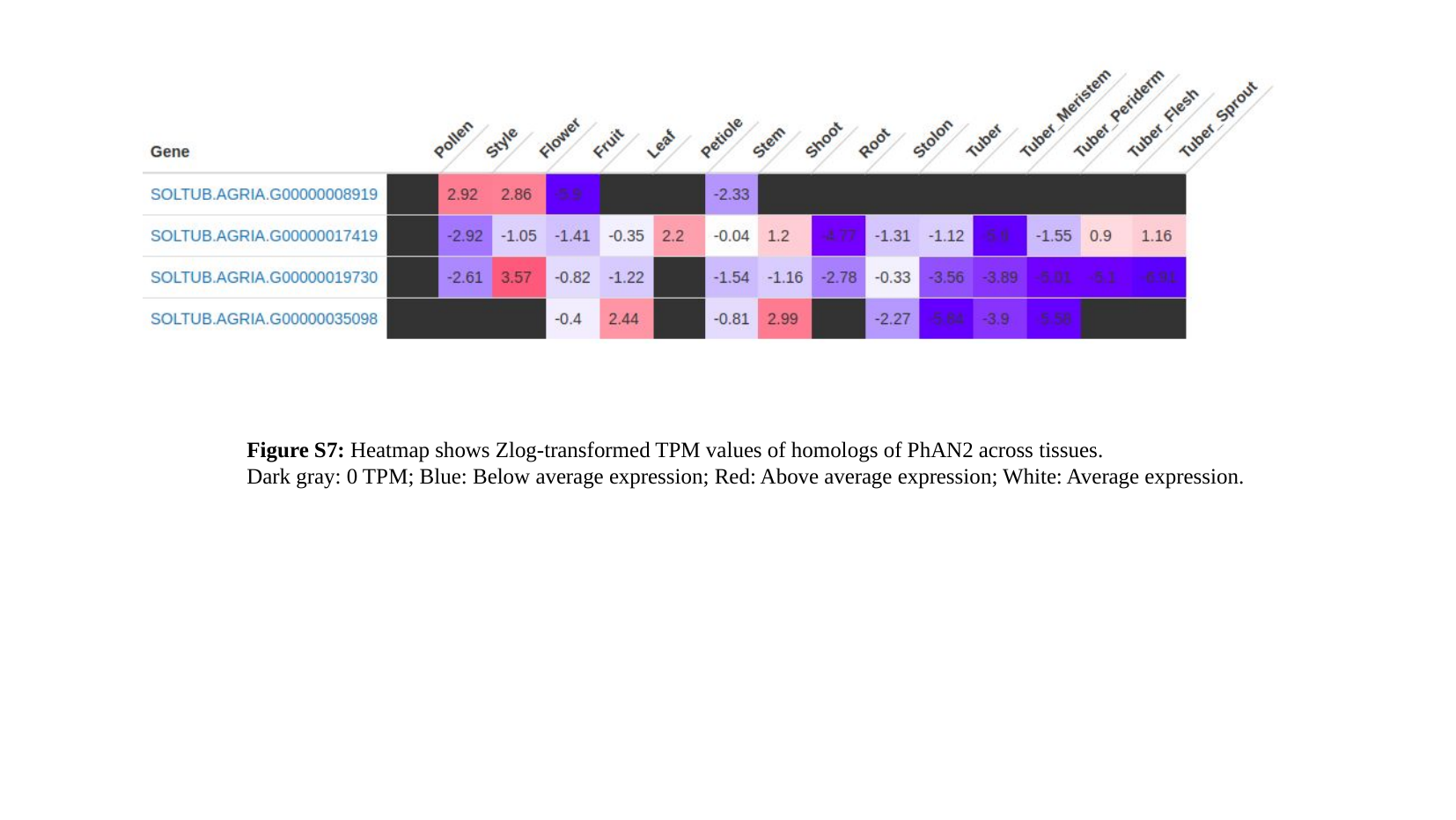

Supplement: Supplementary file 1 — Supplementary file1 (PPTX 829 KB) [file 299_2024_3201_MOESM1_ESM.pptx]
